# Genetic and Regulatory Mechanisms of Comorbidity of Anxiety, Depression and ADHD: A GWAS Meta-Meta-Analysis Through the Lens of a System Biological and Pharmacogenomic Perspective in 18.5 M Subjects

**DOI:** 10.3390/jpm15030103

**Published:** 2025-03-05

**Authors:** Kai-Uwe Lewandrowski, Kenneth Blum, Alireza Sharafshah, Kyriaki Z. Thanos, Panayotis K. Thanos, Richa Zirath, Albert Pinhasov, Abdalla Bowirrat, Nicole Jafari, Foojan Zeine, Milan Makale, Colin Hanna, David Baron, Igor Elman, Edward J. Modestino, Rajendra D. Badgaiyan, Keerthy Sunder, Kevin T. Murphy, Ashim Gupta, Alex P. L. Lewandrowski, Rossano Kepler Alvim Fiorelli, Sergio Schmidt

**Affiliations:** 1 Division of Personalized Medicine, Center for Advanced Spine Care of Southern Arizona, Tucson, AZ 85712, USA; 2 Department of Orthopaedics, Fundación Universitaria Sanitas, Bogotá 110131, Colombia; 3 Department of Orthopedics, Hospital Universitário Gaffree Guinle Universidade Federal do Estado do Rio de Janeiro, Rio de Janeiro 21941-909, Brazil; 4 Department of Orthopaedic Surgery, University of Arizona, School of Medcine, Tucson, AZ 85724, USA; 5 The Kenneth Blum Behavioral & Neurogenetic Institute, Austin, TX 78701, USA; ashim6786@gmail.com; 6 Institute of Psychology, ELTE Eötvös Loránd University, H-1117 Budapest, Hungary; lewandro@usc.edu; 7 Department of Molecular Biology, Adelson School of Medicine, Ariel University, Ariel 40700, Israel; thanos@buffalo.edu (P.K.T.); albertpi@ariel.ac.il (A.P.); bowirrat@gmail.com (A.B.); dr.igorelman@gmail.com (I.E.); 8 Division of Addiction Research & Education, Center for Sports, Exercise, Psychiatry, Western University Health Sciences, Pomona, CA 91766-1854, USA; dbaron@westernu.edu (D.B.); sunderkr@gmail.com (K.S.); 9 Cellular and Molecular Research Center, School of Medicine, Guilan University of Medical Sciences, Rasht 4144666949, Iran; alirezasharafshah@yahoo.com; 10 Behavioral Neuropharmacology & Neuroimaging Laboratory on Addictions (BNNLA), Clinical Research Institute on Addictions, Department of Pharmacology and Toxicology, Jacobs School of Medicine and Biomedical Sciences, University at Buffalo, Buffalo, NY 14260, USA; kyriaki.thanos@buffalo.edu (K.Z.T.); richazirath@gmail.com (R.Z.);; 11 Department of Applied Clinical Psychology, The Chicago School of Professional Psychology, Los Angeles, CA 60601, USA; drnjafariedd@gmail.com; 12 Department of Health Science, California State University at Long Beach, Long Beach, CA 90815, USA; foojanzeine@gmail.com; 13 Department of Radiation Medicine and Applied Sciences, University of California San Diego, La Jolla, CA 92093, USA; mmakale@health.ucsd.edu; 14 Department of Psychiatry, Stanford University School of Medicine, Palo Alto, CA 94305, USA; 15 Department of Psychiatry, Cambridge Alliance, Harvard University School of Medicine, Cambridge, MA 02215, USA; 16 Brain & Behavior Laboratory, Department of Psychology, Curry College, Milton, MA 02186, USA; edward.modestino@gmail.com; 17 Department of Psychiatry, Texas Tech University Health Sciences, School of Medicine, Midland, TX 79430, USA; badgaiyan@gmail.com; 18 Department of Psychiatry, Mt. Sinai University, School of Medicine, New York, NY 10027, USA; 19 Department of Psychiatry, University California, UC Riverside School of Medicine, Riverside, CA 92521, USA; 20 Division of Personalized Neuromodulations, PeakLogic, Del Mar, CA 92130, USA; kevin@prtms.com; 21 Department of Biological Sciences, Dornsife College of Letters, Arts and Sciences, 3616 Trousdale Pkwy, Los Angeles, CA 90089, USA; 22 Programa de Pós-Graduação em Neurologia, Universidade Federal do Estado do Rio de Janeiro, Rio de Janeiro 20270-004, Brazil; fiorellirossano@hotmail.com (R.K.A.F.); slschmidt@terra.com.br (S.S.)

**Keywords:** anxiety, depression, ADHD, autism spectrum disorder (ASD), reward deficiency syndrome (RDS), genes, epigenetics, dopamine dysregulation, GWAS, pharmacogenomics (PGx)

## Abstract

**Background:** In the United States, approximately 1 in 5 children experience comorbidities with mental illness, including depression and anxiety, which lead to poor general health outcomes. Adolescents with substance use disorders exhibit high rates of co-occurring mental illness, with over 60% meeting diagnostic criteria for another psychiatric condition in community-based treatment programs. Comorbidities are influenced by both genetic (DNA antecedents) and environmental (epigenetic) factors. Given the significant impact of psychiatric comorbidities on individuals’ lives, this study aims to uncover common mechanisms through a Genome-Wide Association Study (GWAS) meta-meta-analysis. **Methods:** GWAS datasets were obtained for each comorbid phenotype, followed by a GWAS meta-meta-analysis using a significance threshold of *p* < 5E−8 to validate the rationale behind combining all GWAS phenotypes. The combined and refined dataset was subjected to bioinformatic analyses, including Protein–Protein Interactions and Systems Biology. Pharmacogenomics (PGx) annotations for all potential genes with at least one PGx were tested, and the genes identified were combined with the Genetic Addiction Risk Severity (GARS) test, which included 10 genes and eleven Single Nucleotide Polymorphisms (SNPs). The STRING-MODEL was employed to discover novel networks and Protein–Drug interactions. **Results:** Autism Spectrum Disorder (ASD) was identified as the top manifestation derived from the known comorbid interaction of anxiety, depression, and attention deficit hyperactivity disorder (ADHD). The STRING-MODEL and Protein–Drug interaction analysis revealed a novel network associated with these psychiatric comorbidities. The findings suggest that these interactions are linked to the need to induce “dopamine homeostasis” as a therapeutic outcome. **Conclusions:** This study provides a reliable genetic and epigenetic map that could assist healthcare professionals in the therapeutic care of patients presenting with multiple psychiatric manifestations, including anxiety, depression, and ADHD. The results highlight the importance of targeting dopamine homeostasis in managing ASD linked to these comorbidities. These insights may guide future pharmacogenomic interventions to improve clinical outcomes in affected individuals.

## 1. Introduction

According to the Centers for Disease Control and Prevention (CDC), mental illness can be defined as conditions which affect a person’s thinking, feeling, mood, or behavior. Approximately one in five children in the United States suffers from some form of mental illness [1]. Individuals with an underlying mental disorder (intellectual disability; HP:0001249) often are diagnosed with additional psychiatric disorders such as anxiety, mood, substance use, sleep disturbances, and antisocial personality disorders, making it a large area of importance [2].

Comorbidity is the occurrence of two or more medical conditions in a patient which is determined by a number of factors at play from genetic and biological characteristics to environmental (epigenetic). Comorbidity is associated with poor health outcome, difficulties in health management, and increased health costs. In the United States alone, 80% of Medicare spending is utilized towards patients with four or more chronic medical conditions. This alone is indicative of the high prevalence of comorbidity faced by patients today. Comorbidity is a growing area of research, given that the coexistence of two or more medical conditions can make it difficult to isolate and identify the symptomology of the conditions separately, making the treatment of the conditions quite difficult and sometimes ineffective [3].

In the recent years, psychiatric disorders are among the most common comorbidities identified in patients. Attention deficit hyperactivity disorder (ADHD) specifically is one of the most common intellectual disabilities faced by both children and adults today. ADHD symptoms can include poor concentration, impulsivity, emotional dysregulation, and hyperactivity [4]. Due to its own known symptoms, ADHD can affect and restrict different aspects of an individual’s life. The high co-occurrence of associated manifestations with ADHD such as anxiety and depression can often facilitate the onset of other psychiatric disorders [5]. Given the challenges that the presence of comorbid psychiatric disorders can facilitate in an individual’s life, this meta-analysis examines the comorbidity of ADHD with anxiety and depression and the various treatment options used to alleviate the symptoms presented by these disorders as well as current shared common genetic variants across the brain reward circuitry and epigenetic insults.

### 1.1. ADHD

ADHD is a neuropsychiatric developmental disorder generally characterized by inappropriate behavior, according to age, including inattention, motor hyperactivity, and impulsivity [6]. In a recent meta-analysis, ADHD was found in 3.4% of children in the general population [7]. An estimated 15–20% of elementary school-aged children fit the diagnostic criteria of ADHD. ADHD was also found to be the most frequent psychiatric disorder in children, with an overall global prevalence of 5.2% [8]. ADHD has been estimated to affect 5–10% of children, persisting into adulthood leading to a 4% prevalence among adults [6]. Additionally, one study showed correlations of ADHD with male gender, being non-Hispanic white, divorced or otherwise previously married, and unemployment [9].

In addition to high prevalence, ADHD can persist in adult life with adverse outcomes [8]. ADHD that continues into adulthood is likely to be inherited since it is a familial disorder [7]. In fact, those with ADHD frequently experience low self-esteem throughout their lifespans and lowered self–perception, often causing feelings of inadequacy and incompetence. One feature that seems to be prevalent in ADHD is Rejection Sensitivity Dysphoria, especially in depressed patients with comorbid inattention [10]. The symptoms of ADHD cause a burden on the patient which can lead to the onset of other affective symptoms such as depression, anxiety, low self-esteem, and others [11].

Despite the understanding that ADHD is linked to dysfunction in dopamine (DA) and norepinephrine (NE) neurotransmitters, studies have been conducted that show ADHD to be connected to dysfunctions in many major neurotransmitter systems. This includes serotonin, acetylcholine, opioid, and glutamate neuropathways. All of these neurotransmitters play a role in memory, executive function, and emotional/behavioral regulation. In fact, it is known that ADHD has a high heritability of 74% [2]. GWAS studies show that approximately one third of ADHD’s heritability is due to a polygenic component, meaning the phenotype is comprised of a sum of multiple common variants, each contributing a small effect [12].

The prefrontal cortex (PFC) in the brain is in charge of regulating attention and behavior, especially executive functions. It also plays a role in processing attention, so that we can ignore irrelevant information and coordinate our attention on applicable stimuli. Evidence reveals that individuals with ADHD tend to have weaker prefrontal cortical function. Neuropsychological analyses show that those diagnosed with ADHD have the same impairment on some tasks (such as behavioral inhibition, reward reversal, and working memory) that would be seen in individuals with prefrontal lesions. An explanation for this may be the brain size reduction of the PFC, specifically the dorsolateral PFC, in ADHD patients as shown in the imaging studies. Brodmann’s areas 44, 45, and 46 subserve important executive functions such as memory, judgement and decision making, and behavior regulation, and can be found in this region. The reduction of somatic matter in this area of the cortex can explain the impairments seen in those with ADHD. Images of this study also showed a difference in grey-matter densities inherited from future relatives with ADHD compared to the controls. In subjects with reduced grey matter, inattentive behavior was shown. In addition, studies using functional imaging found deficits in the prefrontal cortex of ADHD patients, including deficits in blood flow and metabolism. These deficits align with poor prefrontal cortical cognitive function [13].

Although there is a genetic factor at play, research has conferred that environmental factors mediate the genetic influences on the outcome and symptoms of ADHD [14]. Environmental factors often play a role in the presence of ADHD. Exposure to various prenatal and perinatal factors of environmental, dietary, and psychosocial natures are attributable to ADHD [7]. A literature review focusing on genetic and environmental causes of ADHD proposed that the strongest factors influencing the effect of ADHD were included fetal alcohol syndrome, pre-maturity, abnormally low birth weight, and behaviors associated with early institutionalized deprivation [15]. These environmental factors can ultimately regulate gene expression (for example, via DNA methylation) and, as discussed below, may open up novel therapeutic targets [16]. It is noteworthy that nicotine usage by pregnant mothers can result in numerous deleterious effects in children, and many of these effects are coupled with obesity. It is indeed possible that individuals carrying ADHD females and smoke during the pregnancy could also carry risk alleles for obesity. It is known that, for example, DRD2 A1 variant may cut across both ADHD and obesity [17]. Theoretical models used to study the effect of nicotine have predicted that the nicotine receptors that modulate dopaminergic activity and dysregulation can cause ADHD. Although the mechanism of how tobacco and nicotine affect the fetal brain remains unclear, it can be hypothesized that perinatal hypoxia may alter neurological development and result in ADHD [14]. A study conducted by Silva et al. in Western Australia identified various risk factors for ADHD onset in diagnosed patients such as stimulant medication for treating ADHD. They found that there was an elevated risk of ADHD in the children of both genders when the mother had a urinary tract infection (UTI) during her pregnancy. Children who were a result of emergency cesarean deliveries, are at greater risk of ADHD later in life. Cord prolapses carried a 2.5 times greater significant increase in the risk for ADHD in female children and breech presentation during delivery of male births was associated with a 17% elevated risk of ADHD. Male children with early term birthday had a 12% elevated risk whereas female children had a 14% elevated risk of ADHD. Low birth weights (1500–2499 g) in male children showed an increased risk for ADHD development. This establishes a clear relationship between child diagnosis and treatment of ADHD in the offspring (irrespective of gender) of: young and single mothers, mothers who smoked during pregnancy, and mothers who experienced threatened preterm labor, preeclampsia, UTI, and induced labor [14].

Furthermore, a long-term follow-up study for children aged 6–12 in regard to childhood ADHD has found that ADHD onset in these participants led to adverse occupational, economic, and social outcomes. An identified increased risk was found in regard to the following: antisocial personality disorder, psychiatric hospital admissions, substance, abuse, incarcerations, and mortality [7]. Studies have found that ADHD is a prominent singular risk factor for substance use disorders even without the presence of comorbid disorder. When comorbid disorders are present, these factors play a key role in increasing the risk of substance use disorder in patients with ADHD [18]. In terms of antisocial personality disorder, Ponce et al. [19] found that the DRD2 A1 allele correlated with increased presence of antisocial personality disorder (60% vs. 15.9%); and a family history of alcohol abuse (72.5% vs. 52.4%). In addition, carriers of the A1+ had an earlier onset of alcoholism and alcohol abuse. This allele is a key factor in the diagnosis of antisocial personality disorder as well as reward deficiency syndrome (RDS).

Research has also shown that the presence of some harmful toxins has been implicated in the etiology of ADHD. Multiple groups of toxins have shown that contamination can contribute to symptoms similar to the ADHD. Lead, manganese, and mercury are considered toxins which can disrupt development and facilitate the development of ADHD pathogenesis. Additionally, prenatal exposure to polychlorinated biphenyls (PCBs) has been associated with manifestations including decreased precision in performance, poor concentration, less attention, and slower reaction time.

### 1.2. Depression

Depression is a serious mood disorder, the presence of which results in symptoms of anger, sadness, worthlessness, suicidal thoughts, frustration, and emptiness, as well as reduced reward functioning. These symptoms can negatively affect the mental, emotional, and physical aspect of an individual’s life. Given the varying severity of the symptoms caused by the onset of depression, it can negatively impact an individual’s ability to function and conduct tasks of daily living [20].

Depression comes in various forms such as persistent depressive disorder, major depressive disorder, postpartum depression, seasonal affective depression, bipolar depression, etc. Major depressive disorder (MDD) can be among the most destructive psychiatric disorders. Symptoms of MDD generally begin during early adolescence, and the prevalence of MDD can increase from childhood levels of some 1% during pre-pubertal years to adult levels of some 6–8% by the end of teenage years. The National Institute of Mental Health estimates that in the United States, 17.3 million adults have experienced at least one major depressive episode. In fact, WHO ranked major depression as the third cause of burden of disease worldwide and projected that the disease will rank first by 2030 [21].

Depression can be categorized by impairment in cognition, emotional regulation, memory, motor function, and neurodegenerative symptoms. MDD, more specifically, is characterized by the following symptoms: persistent and pervasive depression, loss of interest or pleasure, and irritability. MDD has also been correlated with changes in appetite, energy, normal sleeping habits, and ability to focus. Additionally, invasive and excessive feelings of worthlessness, guilt, and preoccupations with death are common in MDD. The presence and symptoms of MDD can have a negative impact on interpersonal and academic functioning [20]. Many individuals whose depression began during adolescence experience its continuation onto adulthood and can suffer from poor outcomes in interpersonal, social, academic, and career functioning in addition to increased risk of substance abuse.

If untreated, MDD can not only cause primary disability but also secondary disability as patients with MDD are compromised making them more likely to develop chronic medical illness [22]. The NIH (2017) found that an estimated 11 million US adults, 18 and older, had at least one depressive episode that caused severe impairment. In fact, within a year, out of all adults who had a depressive episode, 63.8% of those qualified as severe impairment (major depressive episode with impairment among adults).

One hypothesis explaining the pathophysiology of depression is the monoamine hypothesis. This hypothesis proposes the cause of depression to be linked to a change in not only levels of monoamines produced, but function as well. The neurotransmitters most responsible include but are not limited to serotonin, norepinephrine, and dopamine. The serotonergic theory suggests that serotonin metabolites are at a decreased level in patients with MDD. The evidence behind this theory is that use of tricyclic antidepressants (TCA), selective serotonin reuptake inhibitors (SSRI), and serotonin norepinephrine reuptake inhibitors (SNRI) increase brain levels of serotonin, helping to reduce symptoms of MDD in patients [22]. These findings indicate that although increased levels of serotonin are necessary for antidepressant medication effects, the reduction of serotonin alone may not be enough to cause depressive symptoms. The newer and more complete understanding of depression focuses on the potential role of brain anatomy in MDD: including regions such as the nucleus accumbens (NAc; ventral striatum) and its dopaminergic input pathways from the ventral tegmental area (VTA). Together, these anatomical regions are called the mesolimbic dopamine system. Moreover, recent studies reveal that unique behavioral phenotypes can be observed with manipulations of proteins within the NAc–VTA circuitry in rodents. These proteins include CREB, dynorphin, BDNF, MCH, and/or Clock, some of which are directly linked to dopamine regulation and depression. Others have suggested that anhedonia, or reward deficiency, motivation deficits, and reduced energy in the majority of people with depression are contributable to molecular and neurobiological alterations (induced via epigenetics) in reward pathways [23].

### 1.3. Anxiety

Feelings of anxiety can be a normal response to daily stressor and challenging situations; where these feelings begin to converge on the border of anxiety disorder is when those normal feelings turn into excessive fear or worrying without much control [24]. Overwhelming fear and worry can become burdensome, hindering the individual from going about their everyday life. Symptoms of anxiety may present themselves as emotionally as feeling tense, jumpiness, restlessness, irritable, or anticipating the worst or they may present themselves physically as headaches, fatigue, shortness of breath, pounding/racing heart, and upset stomach.

Anxiety disorders are also known to be one of the most prevalent psychiatric conditions affecting both children and adolescents, with a prevalence of up to 5% in adolescents and 3–6% in adults [25]. Anxiety disorders also have a lifetime prevalence of 15–20% in children in the US [26]. The American Psychiatric Association reports that anxiety disorders affect nearly 30% of adults at some point in their lives. Psychiatric disorders, like anxiety disorders, have been found to increase the risk of suicidal thoughts and attempts, increasing risk of mortality and morbidity. They also can lead to substance abuse disorders, depression, and other psychiatric disorders [27].

Additionally, untreated anxiety disorders are linked to the development of secondary psychiatric disorders such as depression, suicidality, and other anxiety disorders. Anxiety can be so debilitating for some that the Epidemiological Catchment Area approximated that about a one fourth of people will experience symptoms so severe that they induce a disability or handicap [25]. Studies examining the neurobiology of anxiety disorders have shown that anxiety is generally caused by a dysfunction in the prefrontal–amygdala circuits [28]. The amygdala is involved in the initiation of central fear responses and has been found to be frequently “overactivated” in functional magnetic resonance imagining (fMRI) studies involving youth diagnosed with fear-based anxiety [27].

Along with the amygdala, the ventrolateral prefrontal cortex (VLPFC) has been examined by many studies in youth with anxiety. The VLPFC regulates amygdala activity and responds with the amygdala to emotional probes. It was noted that the VLPFC is hyperactivated in youth with anxiety disorders, and that it has a degree of activation that is negatively proportional to the severity of symptoms. Because of this evidence, fluoxetine and cognitive behavioral therapy have been found to increase activity of this structure within anxious adolescents [27]. In fact, it is known that early life stress (ELS) is a significant risk factor for psychopathology [29]. Importantly, the literature reveals that ELS is associated with structural and functional modifications of the medial prefrontal cortex (MPFC). Philip et al. showed that compared to controls, the ELS group had significant reductions in default network (DN) connectivity. However further analyses showed a trend-level connectivity enhancement between the MPFC, and amygdala associated with ELS history, suggesting a reduced DN connectivity [29].

### 1.4. Comorbid Anxiety and Depression

In many adolescent cases, MDD is a psychiatric comorbid manifestation with anxiety which is the most common psychiatric comorbidity [30]. A dimensional approach classifies elevated anxiety in major depression as anxious depression [31]. Studies have shown that more than 70% of individuals who are diagnosed with depressive disorders tend to have symptoms of anxiety, and 40 to 70% of them meet the criteria for at least one type of anxiety disorder [32].The presence of anxiety symptoms or disorder tends to complicate the treatment of depressive disorders. Furthermore, those individuals who experience comorbid depression and anxiety tend to be resistant to the standard treatment of antidepressant medication. Individuals with depression tend to become more disabled and dysfunctional when anxiety symptoms or disorders come into effect. Depression was found to be ranked 4th (with anxiety close behind it) among all medical illnesses that contribute to the disabling impact on the world population [32].

Co-occurring anxiety and depression typically present clinically as one of four combinations in patients. First, the criteria for a diagnosis of anxiety disorder may be present with subsyndromal depressive symptoms. Second, the criteria for diagnosis of depressive disorder may be present with subsyndromal anxiety symptoms. Third, a patient might exhibit a complete array of symptoms, leading to diagnoses of both anxiety disorder and depression. In contrast, fourth, a patient could display symptoms associated with both conditions, yet neither could reach the severity threshold for diagnosis. Patients experiencing concurrent depression and anxiety tend to endure more severe illness, longer duration of symptoms, and encounter notably greater challenges in work, psychological well-being, and overall quality of life compared to individuals without comorbidity. Furthermore, clinical risks of depression/anxiety comorbidities include the following: increased risk of psychiatric hospitalization, disability, and suicide, as well as decreased compliance of medical illness and increased used of medical services.

### 1.5. Comorbid Anxiety or Depression with ADHD

The diagnosis of depression is often combined with anxiety disorders, ADHD, and other disruptive behavioral disorders [20]. ADHD is also associated with comorbid psychiatric disorders because they share the same brain regions and neurotransmitter systems that are pathophysiologically implicated [2].

In recruitment of major studies on ADHD, it was seen that severe ADHD has been accompanied by the presence of anxiety, defiance, and disruptive behavior, indicating the strong prevalence of comorbid disorders with ADHD [33]. Compared to adolescents without depression, adolescents with depression face two to three times greater risk for anxiety, attention deficit hyperactivity disorder (ADHD), and behavioral disorders [34].

The two most common psychiatric disorders occurring in young adults are anxiety and ADHD. These two disorders affect about 5% of children at any given time in their life and have a comorbid rate of about 25%. In fact, generalized anxiety disorder (GAD) is one of the most common anxiety disorders in comorbidity with ADHD during both childhood and adolescence; studies conducted in adulthood show that patients with GAD were more likely than adults with social phobia to have a childhood history of ADHD [35]. Separately, both anxiety and ADHD are linked to a great amount of distress and a decrease in social and academic functioning, but the comorbidity leads to a larger functional impairment than either disorder by itself [36].

A study investigating comorbidity prevalence by race/ethnicity found that black adolescents with depression have a significantly higher comorbidity of ADHD (61.1%) compared to Hispanic (43.2%) and white (42%) youth. In adolescents with depression, anxiety prevalence was found to be higher among Hispanics (52.9%) and white (59.2%) youth as compared to black youth (47%). Additionally, the results of the study indicated that regardless of race or ethnicity, depressed adolescents had increased rates of ADHD compared to race/ethnicity matched adolescents who did not have depression [34].

Exposure to both amphetamine (AMPH) and methylphenidate (MPH) result in the increased synaptic availability of DA and NE. However, differences in the specific cellular mechanisms of these drugs may influence their effects on the neurobiological substrates of ADHD and response to treatment in individuals with ADHD. These differences may also influence the drugs’ effects on common comorbidities such as anxiety and depression [2]. AMPH, although generally used to treat ADHD, has an effect on comorbid anxiety and depression as well. Theories have been proposed discussing the possibility that stimulant-associated augmentation of serotonergic drive could improve comorbid anxiety related to ADHD [37].

## 2. Pharmacological Treatment

### 2.1. ADHD

Psychostimulants are the primary pharmacotherapeutic recommendations for children, youth, and adults diagnosed with ADHD treatment; the two most common stimulant medications include MPH and AMPH. The use of stimulants for children diagnosed with ADHD has been correlated with decreases in symptoms including interrupting, fidgeting, and fingertipping, and increased on-task behavior in the classroom. Additionally, stimulants decrease variability and impulsivity in response cognitive tasks, with increased accuracy, and improvements in short-term memory, sustained attention, and reaction time.

Despite the many benefits of stimulant medications, many prominent side effects involved in their use have been documented, including glaucoma, sensitivity to drug response, symptoms of cardiovascular complications, hyperthyroidism, and hypertension. Despite their proven efficacy, there is controversy over the use of psychostimulants for ADHD as there have been many reports of an imminent risk involved of misuse and abuse. Studies conducted using both MPH and AMPH have found a clear relationship between use of these medications and potential for abuse in animal and human models [38]. These studies demonstrate that MPH and AMPH may have reinforcing effects on the brain like other drugs of abuse such as cocaine [39].

Both MPH and AMPH stimulate the mesolimbic dopamine “reward” pathways in the nucleus accumbens, which is a focal point for the potential to abuse. These drugs can be taken in a variety of ways, including intravenous, intranasal, or inhalation, and the pathway of administration could be a key factor in determining the abuse potential of these due to differing rates of absorption and clearance. Furthermore, administration routes have a significant role in regulating the timing and pace of central dopaminergic elevations. When compared to oral routes of administration, intravenous and intranasal routes produce a more rapid increase in central dopamine concentrations. A series of experiments showed that even though oral and intravenous MPH resulted in similar changes in striatal dopamine concentrations, intravenous MPH induced a “high” unlike oral MPH due to the faster rate at which dopamine peaked with intravenous administration [18].

When deciding the best psychostimulant for treatment of ADHD, it is paramount to consider the stimulant, its formulation, and an optimal dose. Concurrently it is necessary to understand the differences in metabolic pathways of the psychostimulants (MPH vs. AMPH) and comprehend the genotype and pathophysiology of the patient [40]. Each individual patient metabolizes the medications differently, leading to differences in patient experience with each stimulant and its respective dosage. More so, most patients go through a guided trial and error phase with their doctor in order to find the right medication and dosing to help properly alleviate their symptoms (see review [41]).

#### 2.1.1. Methylphenidate (MPH)

An ADHD diagnosis does not automatically indicate that pharmacological treatment is the necessary course of action. Treatments vary depending on the degree of the disorder, along with related mental and social limitations that the disorder plays in an individual’s life [5]. In terms of pharmacotherapy, MPH is the most commonly prescribed for ADHD, but it has been shown to have adverse side effects like high potential of abuse and addiction [42].

MPH (Ritalin^®^) is a stimulant that targets the dopamine and noradrenaline transporters in the plasma membrane [40]. MPH inhibits the reuptake of both norepinephrine and dopamine, increasing synaptic availability in the cerebral cortex and hippocampus [42]. Because MPH boosts extracellular dopamine and norepinephrine levels, the heightened efflux of dopamine and norepinephrine results in increased availability of dopamine and norepinephrine to bind with their respective transporters or receptors [2]. For example, 10 mg/kg of MPH administered to spontaneously hypertensive rats produced an increase in the extracellular PFC norepinephrine and striatal dopamine [40]. The dopamine increase helps to stabilize the symptoms of ADHD, including better management of mood, motivation, and attention [7]. One argument in contrary to the utilization of this dual reuptake inhibitor is that increasing norepinephrine in the brain could augment the effects of stress and worsen anxiety.

MPH can have psychomotor effects common to stimulants. MPH blocks the dopamine transporter, resulting in dopamine level spikes in neural pathways that are generally activated by drugs of abuse. These responses (blocking the dopamine transporters by MPH) are dose-dependent. With repeated use, MPH causes long lasting changes in cellular, molecular, and behavioral domains that have been observed in drug abuse. Currently, two forms of MPH are used for pharmacological treatment, one being an immediate-release form and another being a slow-release form. The most commonly prescribed form of MPH is the immediate-release, which is generally prescribed twice a day, given with breakfast and lunch. The sustained-release formula, which is less commonly prescribed given its lower efficacy when compared to the immediate-release, is generally given once daily with breakfast. Adolescent exposure to MPH poses a major concern on public health due to the long-term effects of MPH [43]. Studies show that MPH during pre- and peri-adolescence (periods of development) result in a blunted response to the rewarding stimuli and an augmented state of negative emotion. The latter includes a heightened responsiveness to aversive stimuli. Furthermore, early MPH exposure can lead to longitudinal modifications of the dopamine system of the brain, resulting in attenuated sensitivity to cocaine reward and a decrease in cocaine seeking [44].

Multiple studies have shown common adverse side effects of MPH, including appetite suppression and growth deceleration [7]. Studies have also noted other side effects such as frequent gazing, feelings of sadness, symptoms of anxiety, and displaying closed off behavior. Chronic prescription use of MP has been found to increase anxiety, aggressive behavior, insomnia, depression, and enhance suicidality [42]. In an attempt to manage or reverse these side effects, stimulant holidays may be applied; these include periods where symptom control may be deemed less crucial, such as weekends, holidays, or school vacations, so changes in dose and use are applied as needed [7].

The common dosing of Ritalin used to treat children with ADHD is typically 0.5 mg/kg, administered twice daily. It is predicted that MPH, at oral doses of 0.3 to 0.6 mg/kg, occupies more than 50% of the dopamine transporters in the brain. MPH is slowly metabolized in humans, taking up to 2.5 h, whereas in rats it takes up to only about an hour. Higher doses of MPH cause effects that blunt common locomotor responses to following drug exposure. Low doses of MPH have been shown to promote catecholamine in the PFC, which has been linked to improvement in the delayed alternation performance of rats, whereas high doses diminished delayed alternation task performance. Studies show that too much catecholamine stimulation can decrease prefrontal cortical functioning [13].

The immediate-release formulation (Ritalin) begins at a dose of 10 mg/day and the maximum recommended dosage is 60 mg/day. In the NIMH Collaborative Multisite Multimodal Treatment Study of Children with ADHD (MTA), results showed that patients who received an average dose of 32.8 mg/day of MPH performed substantially better on a range of outcomes as compared to a control group who received a lower mean dosage of MPH of 18.7 mg/day. Because each individual reacts differently to stimulant treatment, the Texas Children’s Medication Algorithm Project recommends starting children on 5 mg/day of MPH with weekly increases until the recommended dosage of 60 mg/day [45].

A study conducted by Volkow and Swanson (2003) established a connection between routes of administration and effects on striatal dopamine concentrations. Volkow found that even though oral and intravenous routes of administration for MPH produced similar changes in striatal dopamine concentrations, oral MPH did not cause a “high” whereas intravenous administration of MPH did. Intravenous administration resulted in much faster rates of dopamine change including peak after 6 to 10 min as compared to oral administration of MPH which yielded a peak after 60 to 90 min [46]. These results lead to the conclusion that, despite other routes of administration which may have more noticeable effects, oral administration of MPH is still a risk for possible abuse. Additionally, studies conducted on the immediate-release (40 mg) of MPH vs. the osmotic release (90 mg) of MPH found that the former induced effects suggestive of abuse potential, such as feelings of a “high” or “drug effects” whereas the sustained-release had only a brief increase in the same subjective effects. The findings from this study emphasize that the overall dosing and formulation of the stimulant are critical in determining the abuse potential of said stimulant [18]. The use of MPH can enhance PFC function in individuals with and without an ADHD diagnosis.

While there is still much to learn regarding the effects of stimulant medications on comorbid anxiety, current studies conducted have concluded mixed findings: acute administration of MPH has been found to reduce anxiety in adults, while chronic treatment beginning early on in life showed an increase in anxiety during adulthood [47]. Meanwhile, some research scientists have postulated that the use of stimulants for ADHD may be in turn alleviating the symptoms of other comorbid disorders; MPH used to treat patients with depression resulted in “moderate or dramatic” improvement in symptoms of depression. This occurred in 10 out of 13 patients, with no serious side effects noted. This is clinically significant given that ADHD has been diagnosed comorbidly with other mental illnesses in patients, allowing one medication to be used for the symptomatic treatment of other comorbid illnesses present along with that of ADHD.

ADHD and substance use disorders (SUD) are often comorbid. ADHD is associated with earlier onsets and more severe SUD. Additionally, SUD treatment effectiveness decreases with ADHD. Screening tools improve diagnostics of ADHD in adults with SUD and should be routinely used and initiated as soon as possible [48]. Simultaneous treatments of ADHD and SUD that combine pharmaco-nutraceutical therapies and psychotherapy must be reconsidered as possibilities [49].

A meta-analysis showed ADHD in childhood is related to adolescent nicotine use and substance use disorders in adulthood. Moreover, Long et al. [50] unveiled shared and distinct brain structural abnormalities among adolescents and young adults with ADHD and SUD. Specifically, reduced grey matter volume (GMV) was noted in the left precentral gyrus, bilateral superior frontal gyrus, and left inferior frontal gyrus in individuals with ADHD compared to control subjects. SUD individuals showed increased GMV in the left putamen and insula when compared to controls. Comparative analysis revealed increased GMV in the right inferior parietal lobule and decreases in the left putamen and left precentral gyrus in the ADHD group when compared to individuals with SUD. Of course, chronic exposure to drugs of abuse can induce brain structural changes [51]. Additionally, the screening of true healthy controls (as described by Long et al.) poses a challenge. It is important that exclusively highly screened controls, free from symptoms and markers of “reward deficiency syndrome (RDS)” should be utilized in all addiction studies [52].

#### 2.1.2. Amphetamine (AMPH)

Short-acting amphetamine (AMPH) can be used to treat ADHD [40]. Generally, those who are unresponsive to MPH find behavioral benefits and improvement from the use of AMPH. AMPH increases synaptic levels of DA and NE, which is done by the inhibition of DA and NE transporters to reduce synaptic reuptake [2].

AMPH’s primary mode of action leads to the release of newly synthesized cytosolic DA from the nerve terminal. AMPH prevents monoamine reuptake by crossing the cell membrane into the terminal and interacting with the transporters responsible for the vesicular storage of DA. Due to this, AMPH transfers DA from the vesicles, leading to an increase in the DA cytoplasmic concentrations; this causes a reverse transport and release from the terminal. The release from the terminal does not require the stimulation of nerve cells and takes place during inactive periods. Additionally, once inside the terminal, AMPH blocks monoamine oxidase, an enzyme that breaks down DA and NE, giving the transmitters more time to be active [39]. AMPH compounds trigger presynaptic dopamine release, leading to an increase in the extracellular DA levels and causing a state of heightened attention and alertness [18]. Increases in DA in multiple brain regions, including the striatum, substantia nigra, and cortex, are credited to the use of AMPH. This psychostimulant has also been shown to modify cerebral blood flow (CBF) to areas such as the striatum, anterior cingulate cortex, PFC, parietal cortex, inferior orbital cortex, thalamus, cerebellum, and amygdala, all dopaminergic areas of the brain. The effects of AMPH on cerebral blood flow are dose-dependent. Low doses decrease CBF in parts of the cortex (frontal and temporal) and striatum. Higher doses lead to CBF increases in the anterior cingulate cortex, caudate nucleus, putamen, and thalamus [2]. Studies have shown that higher doses of AMPH can impact serotonin (5HT) regionally [53].

The most common dose of AMPH for ADHD children is 0.25 mg/kg. AMPH has a 6 to 8 h half-life in humans and about 60 min in rats [53] with a peak observed between 1.3 to 3 h, depending on the dosage. The Texas Children’s Medication Algorithm Project recommends a starting dose of 2.5 mg/day (mixed AMPH salts) with weekly increases up to 30 mg/day of MAS during the first 4 weeks [45]. Commonly, AMPH for ADHD entailed two oral low doses of 0.1 to 0.5 mg/kg taken twice a day, with dosage ranging as high as 0.9 mg/kg/day for best response [54]. Although it has been shown that 40% of children diagnosed with ADHD have equal responses to MPH and AMPH, around 35% of children responded better to AMPH [54]. Evidence has suggested that AMPH may be more potent than MPH [55]. Intraperitoneal administration of 1 mg/kg AMPH to spontaneously hypertensive rates have been found to produce a 15-fold increase in the striatal DA within 30 min of administration, and a normalization within 90 min. Additionally, 1 mg/kg of AMPH produces a 4-fold increase within the NE concentrations in the PFC approximately 45 min post dose and the maintenance of higher-than-control levels for at least 3 h [40]. A study comparing short-acting mixed AMPH salts (MAS) with short-acting MPH, with mean daily doses in the final week of 12.5 mg MAS and 25.2 mg MPH, found that short-acting AMPH was superior to short-acting MPH stimulants at optimal daily doses. Adverse effects of AMPH have been reported to be stomach aches, irritability, appetite loss, tiredness, headaches, and negative emotions such as sadness and tearfulness [40]. Other side effects include “being sad/unhappy” and “prone to cry” [55]. Furthermore, a review on chronic AMPH effects shows that chronic AMPH, even at appropriate doses, may cause substantial variations in the brain’s system and function [56]. It is noteworthy that being female, having conduct disorders present in childhood, and being of older age at the start of treatment significantly increases the risk of later SUD, including alcohol abuse [57]

### 2.2. Depression

Depression is treated by two varied drug classes. One is tricyclic antidepressants (TCAs) which, according to the Mayo Clinic, are cyclic antidepressants that help ease the symptoms onset by depression by blocking norepinephrine and serotonin reuptake [58]. The second class is selective serotonin reuptake inhibitors (SSRIs). SSRIs inhibit the serotonin reuptake transporter (5HT) while weakly inhibiting dopamine and norepinephrine reuptake mechanisms [25]. The mechanism of action of SSRIs works by increasing serotonin in the central nervous system (CNS) [59]. Since its release is no longer accompanied by presynaptic transport back into the neuron, when an SSRI binds to the serotonin transporter, it causes serotonin to accumulate in the synapse.

With more studies favoring SSRIs in terms of tolerability and acceptability, they have begun to replace TCAs and are now the most common medication used to treat depression. In addition, unlike TCAs, SSRIs have a vast therapeutic range and are less likely to have serious anticholinergic effects, including constipation, urinary retention, blurred vision, confusion, and orthostatic effects. The vast therapeutic applications of SSRIs allows for the use of this drug class in the treatment of anxiety and substance abuse disorder along with depression [59].

Adverse effects of SSRIs include mood changes such as agitation and irritability, gastrointestinal distress and appetite changes, headaches, sedation and sleep disturbances, diaphoresis, and sexual side effects. Although suicidal tendencies have been found with the use of SSRIs, the literature has calculated a low risk of suicidality among children and adolescents taking SSRIs. Up to 2% of children have suicidal ideations and behaviors, excluding actual suicides [60]. Furthermore, the first 9 days of treatment pose the greatest suicide risk, especially with higher doses [1]. In another more recent study, a total of 447,411 new antidepressant users were identified. Compared to SSRIs, patients who received SARIs [adjusted hazard ratio (aHR) = 1.124, 95% confidence interval (CI) = 1.108–1.142], SNRIs (aHR = 1.049, 95% CI = 1.033–1.065), and other classes of antidepressants (aHR = 1.037, 95% CI = 1.024–1.051) were more likely to exhibit poor medication noncompliance. Patients who received SNRIs had a higher risk of attempted suicide (aHR = 1.294, 95% CI = 1.114–1.513), compared to SSRIs. However, patents in the TCAs group revealed the opposite result (aHR = 0.543, 95% CI = 0.387–0.762). Concerning the risk of completed suicide, this analysis detected no statistical significance across different types of antidepressants [61].

After utilization, the body attempts to eliminate SSRIs via hepatic metabolism and renal elimination. More than 90% of the original parent compound becomes inactivated [62]. SSRIs with shorter half-lives tend to have increased discontinuation syndromes. This includes nausea, vomiting, abnormal sensations in the extremities, flu-like symptoms, imbalance, sensory disturbances, hyperarousal and sleep disturbances, anxiety, and a worsening of depressive symptoms [63].

Serotonin syndrome is the onset of adverse effects associated with serotonergic medications that increase serotonin in the central nervous system. Serotonin syndrome potentially poses as a challenging side effect of SSRI treatment in adolescents. The symptoms may be mild but can become more severe when coupled with other medications affecting the serotonin central nervous system functioning [64].

For major depression, the two most commonly recommended medications for immediate treatment are fluoxetine (Prozac) and sertraline (Zoloft). Both of these medications have shown considerable improvement in major depression of patients undergoing treatment with additional improvements in comorbid anxiety [31].

The initiation of pharmacological treatment with SSRIs should be done with small doses to recognize the initial target dose for the medication. The medication should then be gradually increased until the patient’s daily dose is the same as the target dose. The initial target dose and increases in dosing must be carefully implemented because an initial too-low dose will not provide sufficient therapeutic efficacy, but one set too high may lead to the presence of many adverse side effects. In addition, increasing the dose too quickly will lead to significant side effects, limiting the patient’s beneficial response [65].

#### 2.2.1. Fluoxetine

Fluoxetine (FLX) is the most common SSRI due to its greater improvement in anxiety and agitation with less troubling adverse effects [66]. FLX enhances central serotonergic transmission. It also has effects on the noradrenergic and dopaminergic systems. Post administration of FLX, serotonergic function is enhanced. The heightened synaptic serotonin synaptic results in both enhanced functioning and decreased activation of presynaptic serotonin neurons. When serotonin uptake is blocked, it builds up in the synaptic cleft upon release. This increases serotonin receptor activation [25].

FLX is best absorbed when administered orally and shows a peak in plasma concentration between 6–8 h [62,65] with a half-life of 4–6 days [25]. The effective dosage of FLX is determined to be 20 to 40 mg daily [65], 60 mg daily [62], or even 80 mg daily [67]. The initial start dose is 5–20 mg, with a target dose for many patients around 20 mg [62,65].

FLX can inhibit its own metabolism and can cause disproportional increases in the plasma levels at high doses. Because of this, physicians should proceed with exceeding caution when prescribing FLX to patients with severe liver and kidney disease or those who are unable to fully utilize their body’s ability to eliminate drugs. While episodic use is not recommended, FLX’s long half-life may be beneficial for adolescents who choose to use FLX medication episodically. This indicates that, even when a dose is missed, there are negligible effects on steady state serum levels. In addition, if adolescents taking FLX have a negative response, enough time should pass for washout before another SSRI is started in order to ward off serotonin syndrome [67].

A study conducted of response to FLX showed that more than 50% of those who will eventually respond will generally show signs of response by week 2 and over 75% will respond by week 4 [68]. Researchers found an additional advantage of FLX in regard to its efficacy; namely that it was the only antidepressant equally effective in children and adolescents. In fact, a meta-analysis including 13 controlled trials of 2910 depressed children and adolescents found that FLX presented a greater pooled efficacy when compared to the other SSRIs used [20]. When compared to sertraline, FLX may induce increased agitation, anxiety, and insomnia [31].

#### 2.2.2. Sertraline

For moderate to severe depression, sertraline may be an optimal first choice [69]. It is postulated that sertraline increases brain serotonin concentrations. When sertraline is administered, it immediately works on the dendrites and axons by blocking serotonin re-uptake pumps in both areas. This blockage leads to an increase in serotonin at the somatodendritic area which causes somatodendritic autoreceptors to downregulate and increase the release of serotonin at the axon. The serotonin increase in the synaptic cleft causes postsynaptic release receptors to downregulate, returning the neurons to their normal state [59].

Sertraline has shown to be best absorbed orally when taken in between meals, with absorption increasing by 1–2 h when taken with food [25]. The recommended dosages are 50 to 200 mg daily, with good clinical outcomes seen at 50 mg daily in many patients. The initial start dose is 25–50 mg with a target dose for many patients around 50 mg. Additionally, the half-life of this SSRI is 24–26 h, with a time to peak plasma concentration between 4.5–8.5 h [62]. Increase in peak concentrations have been seen up to 25% and, when taken with food, peak concentrations have been shown to decrease. Plasma concentrations of sertraline rise proportionally to dose and should be considered as a better SSRI option when dealing with patients who have significant kidney or liver dysfunction [67].

Although sertraline and fluoxetine have had comparable depression treatment efficacy, sertraline was favored in regard to quality-of-life measures and from an economic standpoint in various studies. Sertraline’s lack of drug interactions makes it a better option compared to other SSRIs and its ability to help with the treatment of anxiety disorder allow for it to be a superior course of treatment when dealing with the presence of comorbid disorders [59]. The meta-analysis of 42 studies showed that efficacy favored sertraline over fluoxetine [70].

While sertraline administration has been linked to fewer side effects in comparison to fluoxetine [59], negative side effects have been reported in 10–20% of adolescents, with effects ranging from insomnia, gastrointestinal disturbances, headaches, dizziness, and sexual dysfunction [59,71].

#### 2.2.3. Other Medications

The other SSRIs used for the treatment of depression include citalopram, duloxetine, escitalopram, fluvoxamine, mirtazapine, paroxetine, and milnacipran [71].

Venlafaxine, a selective norepinephrine reuptake inhibitor (SNRI), is another popular medication for the treatment of depression. SNRIs act on both serotonin and norepinephrine, but do not interact with histaminic and cholinergic–adrenergic receptors. This allows for alleviation of negative side effects of medication such as dry mouth, hypotension, and sedation. Despite this, the use of venlafaxine is uncommon due to the presence of suicidal risk that is involved with the intake of the medication that may not be involved with the intake of other medications [20]. When compared to people taking other medications such as fluoxetine (0.4%), previous suicidal behavior in patients taking venlafaxine was 1.0%. Additionally, venlafaxine patients were found to be of higher risk for depressive hospitalization than patients on other antidepressant medications [72].

Bupropion is another common medication which falls under the drug class of norepinephrine dopamine reuptake inhibitor (NDRI). While the mechanism is not entirely known, it is postulated that bupropion works to increase the turnover of NE in the body and blockades the reuptake of DA. Because of this dopamine blocking activity, the literature suggests that bupropion is less likely to be linked to the initiation of manic or hypomanic-like symptoms. Although bupropion has an indicated efficacy in the treatment of depression, it is not generally used as an antidepressant [73].

Paroxetine potently inhibits 5-HT reuptake while blocking the reuptake of NE as well. Citalopram is among the most precisely selective (for the serotonin transporter) compared to most other SSRIs. Citalopram does not have a large first-pass elimination, unlike other SSRIs [25]. These medications are less utilized because of low efficacy and adverse side effects which include diarrhea, dizziness, headache, insomnia, and nausea. In another study, Hansen et al. reported that, on average, patients who had been taking mirtazapine, a non-SSRI, had a 2 kg increase in body weight over 8 weeks of treatments. Additionally, SSRI trial studies reported that paroxetine medication led to the highest changes in body weight [71]. In summary, the increase in use of SSRIs in Canadian children and adolescents from 2005 to 2009 suggests that the effects of public health warnings concerning suicidal thinking and behavior associated with these drugs are now dissipating. This may be attributable to the FDA’s pediatric approvals for fluoxetine and escitalopram, the growing comfort of clinicians with using SSRIs in children, limited availability of psychosocial treatments, and the influence of marketing. The use of paroxetine has continued to decline, likely because of specific warnings directed toward this agent and limited evidence supporting its efficacy [74].

Some of these medications have higher efficacy favoring one SSRI over the other. The meta-analysis of 42 studies yielded results indicating the following: efficacy favored escitalopram over citalopram; citalopram over reboxetine and paroxetine; mirtazapine over and venlafaxine; and venlafaxine over fluvoxamine. Furthermore, results found that escitalopram, mirtazapine, sertraline, and venlafaxine were significantly more efficacious than duloxetine, fluoxetine, fluvoxamine, paroxetine, and reboxetine. Reboxetine had the least efficacy out of all the other medications. Duloxetine and paroxetine showed lower tolerability compared to escitalopram and sertraline, while fluvoxamine exhibited less tolerance than citalopram, escitalopram, and sertraline. Venlafaxine was less tolerated than escitalopram, and reboxetine showed lower tolerability compared to several other antidepressants, including citalopram, bupropion, escitalopram, fluoxetine, and sertraline. Additionally, escitalopram and sertraline demonstrated higher tolerability than duloxetine, fluvoxamine, paroxetine, and reboxetine [70,75]; mirtazapine, escitalopram, venlafaxine, and sertraline were identified as some of the most effective treatments. Moreover, escitalopram, sertraline, bupropion, and citalopram exhibited superior tolerability compared to the other antidepressants. On the other hand, reboxetine, fluvoxamine, paroxetine, and duloxetine were found to have lower efficacy and acceptability, rendering them less preferable options for major depression treatment [70]. Furthermore, the authors observed that mirtazapine, escitalopram, venlafaxine, and sertraline demonstrated higher efficacy compared to duloxetine, fluoxetine, fluvoxamine, paroxetine, and reboxetine. Among the medications, escitalopram, sertraline, citalopram, and bupropion were the most widely accepted [69]. Of note, one fourth of all patients who switched from their initial course of treatment to another SSRI (sertraline), SNRI (venlafaxine), or NDRI (bupropion) reached remission with the second antidepressant, and the overall side-effect problem and rate of serious adverse events did not differ [69].

### 2.3. Anxiety

Selective serotonin reuptake inhibitors are widely prescribed for the treatment of anxiety and are considered to be the first line of pharmacotherapy in adults with GAD [25]. SSRIs have established a proven efficacy, are well tolerated, and have milder side effects compared to other medications [26].

Fluoxetine, sertraline, and paroxetine have all shown efficacy in studies determining their use for anxiety. In a study with adults suffering from generalized anxiety disorder (GAD), both sertraline and paroxetine showed an improvement in anxiety symptoms. Additionally, in a study conducted comparing paroxetine to benzodiazepines, benzodiazepines improved earlier in the course of their treatment while paroxetine was associated with improved symptoms of anxiety. This improvement associated with paroxetine was greater than the anxiety relief associated with benzodiazepines [25].

Citalopram and escitalopram have also been approved by the Food and Drug Administration (FDA) for the treatment of anxiety disorders such as GAD. In three trials of testing escitalopram vs. placebo for the treatment for GAD, escitalopram was found to be superior to the placebo. Duloxetine, a selective serotonin and norepinephrine reuptake inhibitor, is prescribed for GAD, resulting in significant improvements in anxiety symptoms. Duloxetine, an SNRI, received FDA approval for the treatment of GAD in children and adolescents [25,76].

SNRIs such as venlafaxine also have been found to reduce anxiety symptoms, although SSRIs are preferred given their larger scale and more rapid treatment response [25]. Fluoxetine and fluvoxamine were found to be more effective than sertraline and venlafaxine. Additionally, fluoxetine, fluvoxamine, and paroxetine were better tolerated and accepted compared to sertraline and venlafaxine. Among them, fluvoxamine and fluoxetine appeared to be the most effective, with fluvoxamine showing particularly high acceptability. SSRIs, excluding sertraline, were identified as the most effective and well-tolerated medications, while the SNRI venlafaxine demonstrated lower efficacy and acceptability in treating anxiety [77].

Benzodiazepines are considered to be second line interventions in adults with anxiety disorders [25] and are the most common non-SSRI medication [26].They have shown effectiveness in reducing symptoms of anxiety, and a dose-dependent relationship has been found to be associated with tolerance, sedation, confusion, and increased mortality [78]. While the combination of antidepressants and benzodiazepines does indicate a decrease in recovery time from anxiety related symptoms, some studies have found that benzodiazepines themselves do not improve long term outcomes of anxiety. The higher risk of dependence and adverse outcomes complicates the use of benzodiazepines as a pharmacological treatment option [79]. Furthermore, benzodiazepines carry the risk of dependence, sedation, and tolerance along with withdrawal syndromes, making them less optimal choices.

Additionally, the results of a longitudinal study of benzodiazepines and SSRIs indicate that benzodiazepines alone are not effective in the treatment of comorbid depression. The drug itself may be associated with dysphoria, dependence, and can be lethal in overdose, especially when combined with alcohol [80]. Additional side effects of benzodiazepines include fatigue, sweating, jitteriness, and tremors [25].

Benzodiazepines act as allosteric modulators by binding to the Type-A γ-aminobutyric (GABAA) receptor. While there are other benzodiazepines like diazepam and clonazepam, alprazolam stands as the sole benzodiazepine approved by the FDA for treating anxiety disorders like GAD. However, despite the efficacy of diazepam and clonazepam in GAD treatment, physicians often restrict their prescription due to worries about misuse and dependency [25]. Alternatively, the genus *Passiflora incarnata Linnaeus* comprises approximately 520 species belonging to the Passifloraceae family. The majority of these species are vines found in Central or South America, with rare occurrence in North America, Southeast Asia and Australia. The genus *Passiflora incarnata* has long been used in traditional herbal medicine for the treatment of insomnia and anxiety in Europe, and it has been used as a sedative tea in North America [81].

#### 2.3.1. Alprazolam

The literature review has shown alprazolam to be significantly superior compared to placebo at providing relief of depressive symptoms. Additionally, despite SSRIs being the first line of treatment, alprazolam is the recommended second line of treatment when SSRIs are not well tolerated or are ineffective at treating symptoms caused by underlying psychiatric disorders such as anxiety [82]. Alprazolam has been observed to impact dopaminergic function in the striatum in a manner like stimulants. Administration of this medication has been associated with a notable rise in extracellular dopamine concentrations in the striatum, along with a noticeable trend towards increased serotonin levels [83].

Alprazolam readily penetrates the blood–brain barrier, gaining access to the CNS where it binds to the GABAA benzodiazepine receptor complex. At this site, alprazolam facilitates the binding of GABA, which leads to inhibition of activity in various interconnected brain regions, resulting in sedation or a reduction in brain activity. Alprazolam also affects serotonergic and noradrenergic pathways extending to both the limbic system and brainstem, which contributes to its efficacy in treating anxiety and depression. Brain structures involved in memory, such as the cerebellum, hippocampus, amygdala, and cerebral cortex, exhibit a high affinity for alprazolam due to their GABAA benzodiazepine receptor complexes. Several reviews on benzodiazepines and memory have highlighted a dose-related impairment of learning and memory, while previously learned information before drug administration remains intact [82].

Generally, in the drug class of benzodiazepines, alprazolam is the most commonly used given its fast onset of symptom relief [82] and because of its lower incidence of sedation [84]. A comparison of oral and sublingual routes of administration found that peak plasma levels were reached earlier with oral administration (1.8 h) when compared to sublingual administration (2.8 h). The metabolism of alprazolam primarily occurs in the liver. Consequently, in patients with liver cirrhosis, the clearance of alprazolam is notably diminished, resulting in an increase in the elimination of half-life of the drug. Similarly, renal diseases can also reduce alprazolam clearance, leading to the accumulation of the medication and its metabolites in the body [82].

Immediate-release (IR) tablets of alprazolam are clinically effective for 3 to 6 h, leading to the administration of the medication occurring multiple times throughout the day, which may affect clinical efficacy due to the fluctuations in plasma levels. There is also a sustained-release (SR) form for alprazolam which reduces inter-dose variability in plasma levels of alprazolam and its adverse effects. The SR form has lower absorption rates than the IR, with peak plasma concentrations at half of the IR form of alprazolam [82].

Studies have shown that the use of alprazolam may reduce panic attacks within a few days of use at the recommended dose of 1.5 to 10 mg, with side effects including drowsiness, fatigue, slurred speech, poor concentration, hypersensitivity, irritability, amnesia, sedation, and memory problems [83]. Alprazolam tends to be more toxic in cases of overdose and has consistently demonstrated a comparable level of anxiolytic effectiveness to other benzodiazepines. Moreover, withdrawal symptoms, notably severe rebound anxiety, are more frequently reported with alprazolam use, primarily attributed to its shorter half-life and higher potency [83].

Alprazolam is generally prescribed to treat panic and anxiety disorders [42]. In a review of the efficacy of alprazolam as a single treatment for anxiety, panic disorder, and depression, researchers found alprazolam to be superior to placebo and as effective or superior than all comparative benzodiazepines, antidepressants, and buspirone [83].

Due to the negative withdrawal symptoms caused by the discontinuation of alprazolam tapering, steps are taken to reduce rebound symptoms including decreasing dosage gradually to 0 mg. Alprazolam is a commonly abused drug given its dose-dependent sedation and feelings of euphoria [82].

#### 2.3.2. Methylphenidate Combination Therapy

A study conducted by Bolanos, Willey, et al. examined the combined effects of FLX and methylphenidate (MPH) and found that exposure to MPH during preadolescence could affect responses to both rewarding and aversive stimuli in adulthood. These behavioral alterations were reversible with subsequent antidepressant treatment later in adulthood.

The study revealed that MPH exposure during preadolescence led to a decrease in normal sensitivity to rewarding stimuli such as sucrose (a natural reward) and morphine during adulthood in rats. This reduced sucrose preference was likely due to MPH’s ability to modify responsiveness to the rewarding effects of the solution. Furthermore, MPH treatment appeared to increase rats’ susceptibility to anxiety and stress-inducing situations. Rats pre-exposed to MPH exhibited heightened reactions to anxiogenic stimuli, spending less time in the open arms of an elevated plus maze and significantly more time engaged in self-grooming, which is a behavioral response to anxiety-inducing situations Moreover, the study indicated that antidepressant treatment could normalize depression-like behaviors following preadolescent exposure to MPH [85]. MPH works by blocking dopamine and norepinephrine transporters in the brain. At low doses, MPH improves cognitive functions without locomotor activating effects, whereas at high doses, MPH may induces hyperlocomotion [86].

In a study investigating the combined effects of alprazolam and MPH on neurobehavioral, biochemical, and histological changes, it was concluded that caution must be exercised in co-administering both drugs due to their adverse effects. High doses of alprazolam, MPH, and their combination were observed to impair Purkinje cells. These cells exhibited shrinkage and damage to their surfaces, resulting in a loss of their typical pyriform shape and a transition to a more triangular shape. Additionally, the molecular and granular layers of the cells appeared compacted and clustered, indicating severe injury and disruption of the normal cellular arrangement.

Co-administration of alprazolam significantly reduced the anxiety-like behavior induced by MPH. However, chronic administration of both drugs, either alone or in combination, led to a significant increase in lipid peroxidation and a decrease in endogenous antioxidant enzyme activities in the cortex and hippocampal regions. Elevated levels of inflammatory markers and oxidative stress indicated neurotoxicity in the hippocampus and cortex, resulting in cognitive impairment. Furthermore, animals exposed to high doses of both alprazolam and MPH showed increased levels of dopamine and decreased levels of hippocampal serotonin.

The administration of alprazolam, both alone and in combination with MPH, resulted in impaired memory as assessed by the novel object recognition test. In this test, rats underwent two trials: in the first trial, each rat was placed in the center of an open chamber containing two identical objects positioned at opposite corners. Three hours later, the second trial commenced, during which the rats were allowed to explore a novel object along with another object similar to it for 5 min. A discrimination index was then calculated to assess the animal’s ability to differentiate between the novel object and the similar object. This test serves as an effective means to evaluate the animal’s short-term working memory and anxiety levels.

In this case, the impaired working memory of the rodents in the novel object test was said to be attributed to the increased activity of acetylcholine in the hippocampus region of the brain. Acetylcholine is an essential neurotransmitter that plays a vital role in learning and memory consolidation. The increased availability of acetylcholine leads to rapid depletion of acetylcholine levels in the synapse, causing a hinderance in the learning and memory consolidation process.

On the other hand, MPH alone produced the opposite effects to that of alprazolam, indicating its potential to induce anxiety associated with increased risk-taking behavior. MPH did increase alertness, explorative behavior, and was not found to alter the acetylcholine levels in the brain [42]

#### 2.3.3. Benzodiazepines Combination Therapy

Benzodiazepines in combination with SSRIs are generally the primary choice for pharmacotherapy in patients with comorbid anxiety and depression [84]. Combination therapies offer significant benefits for individuals experiencing symptoms consistent with multiple mental health disorders, such as co-occurring anxiety and depression. SSRIs are typically recommended as the first line of treatment due to their proven effectiveness, wide-ranging activity, generally favorable tolerability, efficacy in addressing concurrent depression, and suitability for prolonged use.

Augmenting an SSRI with a benzodiazepine can lead to greater improvement in depressive symptoms and stabilization of panic or social phobia symptoms compared to using SSRIs alone. This combined approach provides enhanced therapeutic outcomes by targeting multiple facets of the individual’s mental health challenges. The advantage of adding benzodiazepine to a pharmacological treatment approach is that benzodiazepine has been shown to have rapid onset and action, providing in efficacy in reducing feelings of anxiety [80]. The combination of these two can provide the patient with anxiety control, a decrease of SSRI-induced anxiety or agitation, improved adherence to antidepressant therapy, and improvements in symptoms of situational anxiety [87]. Although the use of benzodiazepine alone may have a depressant effect on a patient, the concomitant therapy of benzodiazepine with an antidepressant can prevent or help alleviate benzodiazepine-related depression. In a meta-analysis of nine controlled depression studies, it was found that patients treated with combination therapy (antidepressant and benzodiazepine) were 37% less likely to discontinue treatment. Furthermore, 38% of the patients were more likely to have more than 50% decrease in baseline depressive symptoms at week 4 when compared to those taking antidepressants alone [66].

## 3. Psychosocial Treatment

When addressing ADHD with pharmacological treatment, it is crucial to incorporate behavioral interventions like optimized classroom management strategies, parental psychoeducation, and behavioral management techniques. These non-pharmacological interventions, particularly behavioral ones, are integral parts of treatment guidelines. Initial findings from the largest trial of ADHD interventions suggest that multimodal treatment involving intensive behavioral therapy alongside pharmacological treatment did not offer additional benefits over pharmacological treatment alone in managing the core symptoms of ADHD. However, studies have indicated that combining psychosocial treatments with medication may yield benefits in terms of managing associated symptoms, improving levels of functioning, and potentially reducing the required medication dosage [7].

On the other hand, three significant multisite, placebo-controlled studies have found that the results of combining SSRI use and psychotherapy to be superior compared to stand-alone treatments for depression, obsessive–compulsive disorder (OCD), and anxiety [1]. The employment of concurrent psychotherapy with pharmacotherapy allows for the alleviation of symptoms linked to primary disorder, but also for the alleviation of negative side effects brought on by the use of pharmacotherapy [25].

### 3.1. Cognitive-Based Therapy

While pharmacologic treatments may be the preferred treatment method for ADHD due to their effectiveness, cognitive-based therapy (CBT) is considered the top choice for treating a wide range of comorbid anxiety disorders occurring in children [36]. Results from clinical trials analyzing medication prescribed for ADHD provide evidence that approximately one out of four patients are either intolerant towards the medication or will not adequately respond to it. Even after seeing improvement in ADHD symptoms from using medication, changes in lifestyle may still be needed for enhancement in various aspects of quality of life [88] and refinement in both self-esteem and self-perception. CBT has been found to even beneficial in the treatment of depression: Paykel et al. found that CBT reduced relapse rates for patients who had residual depressive symptoms [89].

CBT is thought-content focused and promotes a different way of analyzing painful and challenging circumstances that one faces. This kind of therapy helps to differentiate dysfunctional and negative thoughts from healthy and positive ones. Furthermore, CBT aims to challenge and test dysfunctional beliefs by working to make new interpretations for them. The behavioral interventions conducted with this therapy are focused on reinforcing adaptive responses [90]. CBT treatment has been demonstrated to be highly effective in both individual and group settings for adults suffering from ADHD. In fact, studies have shown that the addition of CBT for patients with limited response to medication may lead to improved outcomes [88].

Evidence from blind randomized control trials conducted to examine the beneficial effect of behavioral interventions on parenting and child conduct problems suggest that CBT may be useful for adults with ADHD when used in conjunction with pharmacological treatment [7]. While children might be placed in therapy at the request of their parents/guardians, adults are self-referred, which may indicate a greater desire for improvement in symptoms. Additionally, upon entering adulthood, patients have had more experience with their ADHD and begin to have a better understanding of its negative implications in their life. This may make them more receptive and motivated to learn about and try different treatments that can help them better cope [88].

Weiss et al. supported previous findings that adults with ADHD are responsive to CBT intervention, leading to improvements in core symptoms and functioning, both with and without the administration of pharmacological therapy [88]. More specifically in regard to children, a study conducted by Gould et al. saw substantial improvement in ADHD symptoms of children after they had gone through CBT for their anxiety, with improvement in symptoms remaining evident at their 6 month follow-up [36]. The improvements in those with ADHD found in this study are consistent with findings of another study which found a 69% ADHD remission rate after CBT, with clear improvements in symptoms at their follow-up appointment. Based on the results, Kendal et al. inferred that those who presented signs of remission involving primary anxiety were more likely to also show signs of comorbidity remission, further concluding that the improvement in primary disorder was linked to improvement in the comorbid disorder as well. CBT has also been denoted as having a “preventative” effect in five out of eight studies. On average, it was found that only about 30% of patients who underwent CBT relapsed as compared to 60% of patients who relapsed when treated with only antidepressants [91].

Although CBT is effective at treating symptoms of ADHD, anxiety, and depression, it has been reported that this treatment is best when combined with pharmacological treatment. Both Safren et al. and Emilsson et al. concluded that CBT in conjunction with stimulants were significantly more effective than stimulant medication alone in reducing the core symptoms of ADHD [92,93]. Additionally, a study conducted by Klein and Abikoff found that stimulant/behavioral therapy combination treatments were significantly superior compared to behavioral therapy alone [33]. This is because when both therapies are combined, they are able to target and improve various aspects of patients’ symptoms. Pharmacological treatment works to alleviate symptoms of hyperactivity, inability to pay attention, and emotional instability, while CBT works to better social functioning and organizational behavior [4]. Furthermore, patients who underwent CBT for ADHD found a substantial improvement in symptoms of comorbid anxiety and depression as well [93].

### 3.2. Mindfulness-Based Cognitive Therapy

While CBT focuses on changing or altering the thoughts of a patient, mindfulness-based cognitive therapy (MBCT) works to help patients understand their relationship to their thoughts and feelings to better increase metacognitive awareness [90]. MBCT is more focused on thought process and works to introduce a new way of coping and living with painful and challenging circumstances by helping patients understand that their thoughts are simply fleeting ideas and not necessarily factual statements. It works by disciplining the individual to focus on noticing and allowing their thoughts and feelings to occur without trying to fix, change, or avoid them. These behavioral interventions focus on developing present moment awareness and becoming more conscious of thoughts throughout the day by increasing the metacognitive awareness in patients. This is accomplished by motivating patients to adopt a unique mode of being which allows the mind to understand the difference between how one views things vs. how they actually are and working to reduce that discrepancy [90].

In MBCT, patients learn that attempting to resist or avoid experiencing unwanted thoughts or feelings may actually intensify distress and lead to the continuation of depression, rather than alleviating it. Behavioral elements are introduced into therapy to support patients and enhance their well-being while working on staying mindful and conscious of one’s thoughts. By developing an action plan to help identify warning thoughts or feelings that may indicate a decline in mental state or worsening symptoms, patients can be prepared to deal with unwelcome thoughts and can initiate steps to alleviate said decline or worsening symptoms.

An essential aspect of MBCT involves enhancing the capacity to intentionally redirect one’s attention, enabling more flexible cognitive and behavioral responses. The functional impairment associated with depression and anxiety disorders often stems from an overemphasis on attending to and attempting to manage unwanted thoughts or emotions, at the expense of other meaningful activities. Through heightened metacognitive awareness, MBCT encourages individuals to actively label unwanted thoughts or emotions as mental events, thereby reducing their perceived threat and requiring fewer cognitive resources to manage. This process enables distressing cognitions to be perceived as less overwhelming, allowing individuals to engage more effectively with their experiences [90].

MBCT can also help alleviate many symptoms of ADHD by focusing on regulating emotions, distress, impulsivity, and attention, along with comorbid anxiety and depression [11]. In terms of active depression, studies conducted on MBCT have found that using MBCT as a therapy for the active phase of recurrent depression showed an improvement in the Beck Depression Inventory (BDI), which is a self-reporting questionnaire designed to evaluate the severity of depression in individual scores at 3 months, with a reduction in the mean score from 38 to 28. They also found an overall improvement in anxiety and a larger degree of tolerability [94]. Additionally, another study with participants who had residual symptoms between acute depressive episodes found that MBCT lead to a significant improvement in BDI scores [95].

Since dopamine is an important neurotransmitter which is involved in both depression and anxiety, our laboratories have repeatedly suggested that one non-invasive safe and non-addictive way to address ADHD issues is to induce ‘dopamine homeostasis”. Interestingly, this difficult task has been accomplished via a number of peer reviewed studies including triple- and double-blind controlled placebo with KB220 variants. The studies include neuroimaging and even stress measured objectively via skin conductance, in naive and SUD (e.g., psychostimulant abuse, alcoholism, and heroin dependence) in both animal models and humans [96,97], and precursor amino acid loading improves inpatient treatment of alcohol and polydrug abusers, as shown by a double-blind placebo-controlled study of the nutritional adjunct SAAVE [98,99,100].

### 3.3. Awareness Integration Therapy

Awareness integration therapy (AIT) represents a multi-modality psychotherapeutic paradigm designed to augment self-awareness, alleviate past traumas and psychological barriers, and foster clarity and positive attitudes. Constructed by amalgamation of insights and techniques drawn from diverse therapeutic models such as cognitive behavioral therapy (CBT), existential therapy, person-centered therapy, emotion-focused therapy (EFT), mind–body therapy (MBT), eye movement desensitization and reprocessing (EMDR), hypnosis, and mindfulness, AIT offers a comprehensive approach. AIT ensures the optimization of therapeutic efficacy, enabling the generation of lasting and transformative outcomes for individuals undergoing treatment [101,102]. AIT acknowledges the individual’s relationship with their substance of choice and their relationships, work, finances, and self-identity [103]. However, relying solely on avoidance of pain often leads to existential resentment and depression, perpetuating a cycle of relapse. Love and acceptance can serve as powerful catalysts for initiating sobriety, yet sustained recovery requires individuals to engage in internal work to address underlying emotional turmoil. AIT empowers individuals to evaluate their thoughts, feelings, and behaviors, fostering self-awareness and facilitating meaningful change, even amidst genetic predispositions and epigenetic influences [104]. AIT has demonstrated efficacy in addressing childhood traumas and reducing rates of depression and anxiety among Children of Alcoholics (COAs). Most individuals with substance dependence, as well as those without, often originate from dysfunctional family backgrounds, with their addictions exacerbating the existing dysfunction. Genetic testing of such families would likely reveal a high genetic predisposition to addiction based on reward gene polymorphisms. COAs, for instance, represent a special population group that could benefit from early genetic screening due to their heightened risk [104]. COAs commonly endure depression, anxiety, and social difficulties, lacking secure role models to navigate life’s challenges. Studies have shown a significant association between COAs and the DRD2 A1 allele, underscoring the genetic vulnerability within this population [105,106]. Adolescent depression and anhedonia are prevalent yet often overlooked and untreated. Approximately one in five children may experience emotional, behavioral, or mental health issues, with a significant proportion suffering from mild to severe depression. However, only a fraction of affected adolescents receives adequate treatment, leaving many vulnerable to substance abuse, risky behaviors, poor academic performance, and even suicide [107]. Given the frequent comorbidity of addiction with mood, anxiety, PTSD, and personality disorders, long-term weekly psychotherapy is imperative. Research on AIT has shown promising outcomes, including significant reductions in depression and anxiety levels, along with improvements in self-esteem and self-efficacy [105]. Given that addiction affects various aspects of an individual’s life, the treatment approach should similarly encompass all these dimensions. Relapse prevention will be facilitated through balancing and creating fulfillment in all areas of life.

In addition to traditional treatment options such as psychostimulant therapy and behavioral approaches, we propose a novel tool called awareness integration (AI), developed by one of our team members (F.Z.). AI represents a pioneering model in psychotherapy, drawing on various techniques from cognitive, emotional, behavioral, and body-mind theories. The primary objective of AI is to enhance self-awareness and self-esteem while addressing past traumas and psychological barriers. Moreover, AI seeks to alleviate symptoms of anxiety and depression, fostering a clear, realistic, and positive mindset, along with equipping individuals with skills for leading effective, productive, and functional lives. AI incorporates flexible questioning techniques and comprehensive interventions that address core beliefs, emotions, and the physical locations where emotions and memories are stored in the body. A pilot study on AI demonstrated promising results, including a 76% reduction in depression, a 60% decrease in anxiety, a 43% increase in self-esteem, and a 20% enhancement in self-efficacy. These findings highlight the potential efficacy of AI as a therapeutic approach for addressing various mental health concerns.

Currently there is real need to understand the epigenetic impact the environment has on comorbidity issues as expressed in this review and meta-analysis article. The literature in general is exploding, especially with ADHD as just one example [12,108,109,110,111,112,113,114,115,116]. In addition, there is a lack of knowledge regarding the actual potential genetic pathways and interaction of these genes in a network leading to the expression of the endophenotype related to the comorbidity of anxiety, depression, and ADHD. To that end we have performed a novel GWAS meta-meta-analysis through the lens of a system biological and pharmacogenomic perspective in 18.5 M subjects.

## 4. Material and Methods

### 4.1. Raw Data, Subjects, and Meta-Data

GWA studies were selected for obtaining raw data. To achieve this laudable goal, all the included information was extracted from the GWAS catalog (available at: https://www.ebi.ac.uk/gwas/home, accessed on 3 November 2024). Initially, the main key words, including “Anxiety”, “Depression”, and “ADHD”, were separately searched and the exactly-matched datasets were downloaded (Raw Datasets). Accordingly, EFO_0005230 was found for anxiety; and EFO_0006788 was also found for anxiety disorder; MONDO_0002050 was found for depressive disorder; and EFO_0003888 was found for ADHD. Each of these datasets contained several GWAS annotations related to this comorbidity topic. Thus, we categorized the anxiety GWAS dataset as Meta1, anxiety disorder GWAS dataset as Meta2, depressive disorder GWAS dataset as Meta3, and ADHD GWAS dataset as Meta4. We also performed four separate meta-analyses for each of the four datasets and a final meta-meta-analysis conducted on all four meta results.

### 4.2. Meta-Analysis and Meta-Meta-Analysis

A meta-meta-analysis refers to the analysis of multiple meta-analyses together to find the cumulative effect size and the significance of assumed clinical manifestations (i.e., anxiety, anxiety disorder, depressive disorder, and ADHD). Specifically, following calculating each GWAS dataset in a comprehensive meta-analysis (CMA) via Comprehensive Meta-Analysis Version 3.3.070 software (Biostat, Englewood, NJ, USA), we obtained meta-data including Meta1, Meta2, Meta3, and Meta4. These data were separately analyzed by CMA3 in accordance with the refined GWAS datasets. It is noteworthy that each GWAS dataset met the inclusion and exclusion criteria. Transparently, the inclusion criteria followed included: unique GWAS, published papers, GWA studies with specific number of cases and controls, a very stringent level of statistical significance *p*-value < 5 × 10^−8^, having OR and CI95%, papers which reported specific mapped genes and Single Nucleotide Polymorphisms (SNPs), The exclusion criteria were as follow: Duplicated studies, unpublished papers (pre-print or under review formats), pilot GWA studies (no separated cases and controls), *p*-value > E−08, lost OR and CI95%, papers lacking specific gene names and SNPs. Meta-analyses and meta-meta-analyses were conducted on the CMA3 tool based on effect size data set up (i.e., two groups or correlation > dichotomous > unmatched groups > sample size and *p*-value). The entry data were as follows: sample size, independent groups *p*-value, 2-tails, positive effect direction (due to GWAS association results).

### 4.3. Systems Biology and In-Depth Silico Analysis

A GWAS meta-meta-analysis was performed for four phenotypes designed on the basis of the GWAS catalog database (https://www.ebi.ac.uk/gwas/home, accessed on 3 November 2024) [117] to uncover all related loci via various Catalog IDs (CIDs). Every CID contains single or multiple GWA studies itself. Following meta-meta-analysis, extracting the mapped genes from each CID, a filtering strategy of refinement was introduced whereby all genes were then combined together. A massive raw file was then constructed that contained unfiltered duplications, RNA genes, pseudogenes, and protein-coding genes. Discarding the duplications and after deleting the RNA genes and pseudogenes, the remaining protein-coding genes were refined. Subsequent further analyses were performed on this final refined file. All of the details and references are summarized in Table 1.

**Figure 1:**
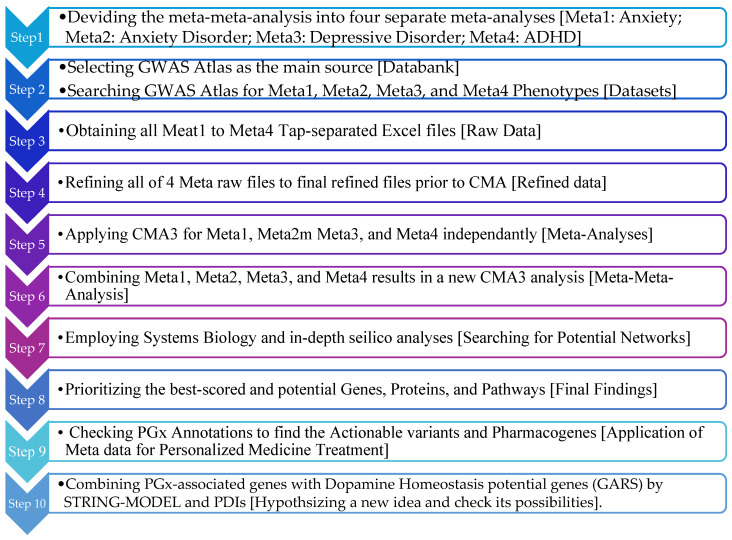
Flowchart of strategy used in this GWAS meta-meta-analysis from the databank selection to the final findings through 10 specific steps. GWAS: Gnome-Wide Association Studies; CMA: comprehensive meta-analysis; PGx: pharmacogenomics; GARS: Genetic Addiction Risk Severity; PDIs: Protein–Drug interactions.

A newly introduced validated strategy based on our previously published papers was utilized to obtain a systems biological approach linked to the refined gene list. For clarity, this additional approach was employed to provide new insights into the comorbidity of anxiety, depression, and ADHD. Our rationale herein was to uncover top candidate genes via the GWAS meta-meta-analysis. We further reasoned that potentially adding the systems biological approach might reveal a higher level of psychophysiological manifestations resulting from both genetic and epigenetic interactions. Moreover, this was accomplished by generating a multi-step investigation specifically linked to the refined final gene list. This included Protein–Protein Interactions (PPIs) by STRING-MODEL, enrichment analysis (EA) by Enrichr, and clustering enrichment analysis (CEA) by Metascape. Additionally, to further display potential personalized medicine treatment for future medical and experimental guidance, we conducted a pharmacogenomics (PGx) investigation. We accomplished this aim using an additional PGx tool known as Variant Annotation Assessments (VAAs) highlighting the importance of our novel refined gene list. As such we believe that the following results could provide healthcare professionals in the future with a genetic/epigenetic map to assist in the therapeutic care of patients presenting with multiple manifestations (anxiety, depression, and ADHD) (see Table 1 and Figure 1).

Figure 1 illustrates a high-level flowchart showing our step-by-step strategy pipeline with a brief description of all steps. This figure emphasis the rationale behind each step in a hierarchical flow from GWAS raw data to the final refined file of potential genes from all the meta-data (Meta1–Meta4).

## 5. Results

### 5.1. Data Refinements and Curation

Prior to our meta-analysis assessment, all four datasets (Meta1, Meta2, Meta3, and Meta4) required refinement and met the inclusion/exclusion criteria. Meta1 had eleven unique GWAS; among them, eight GWAS passed the inclusion/exclusion filtering process. The final sample size of Meta1 was 794,939. Meta2 had 47 unique GWAS; among them, 23 GWAS passed the inclusion/exclusion filtering process, and the final sample size of Meta2 was 4,344,931. For Meta3 and Meta4, the initial GWAS were 17 and 44 and refined to 15 and 28, respectively. Table 2 summarized the refined data that was used for separate meta-analyses and meta-meta-analysis.

Following refining the Datasets of Meta1, Meta2, Meta3, and Meta4, raw data indicated that there are 18,468,527 subjects in 74 studies reporting 1269 associations among various ethnicities including European, African American, Native American, Latino American, Asian, East Asian, South Asian, and Hispanic, and specifically Han Chinese, British, Korean, Danish, Russian, and Japanese people. Of note, European had the majority portion of total sample size (Table 2).

### 5.2. Meta- and Meta-Meta-Analysis Results

**Figure 2:**
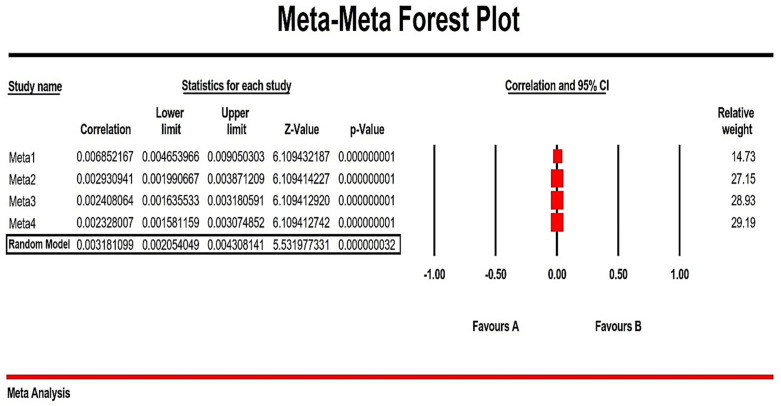
Forest Plot of GWAS meta-meta-analysis based on four separate meta-analyses including Meta1 (anxiety), Meta2 (anxiety disorder), Meta3 (depressive disorder), and Meta4 (ADHD).

We successfully carried out four separate meta-analyses for each GWAS dataset with the CMA3 tool. We employed this meta- and meta-meta-analysis to determine final effect size of GWAS in each dataset (Meta1, Meta2, Meta3, and Meta4) based on Forest Plot and random model of effect. CMA3 indicated that the statistical scores for Meta1 were as follows: *p*-value < 1E−10; OR = 0.026 [0.020–0.032]; Z-value = 8.164. Meta2 also represented a *p*-value lower than 1E−10; OR of 0.023 [0.020–0.027] and a Z-value of 11.544. For the other two datasets, the results were as follow: *p*-value < 1E−10; OR = 0.020 [0.016–0.025]; Z-value = 8.99 (Meta3); *p*-value < 1E−10; OR = 0.026 [0.022–0.030]; Z-value = 12.994 (Meta4) (Figures S1–S4). All the details for publication bias (Funnel Plots) (Figures S5–S8) and models of statistical calculations will be available on a reasonable request from corresponding author. Final results of meta-meta-analysis for Meta1, Meta2, Meta3, and Meta4 in a random model of Forest Plot were as follows: *p*-value = 3.2E−08; OR = 0.003 [0.002–0.004] and Z-value = 5.532 (Figure 2).

### 5.3. Systems Biology and In-Depth Silico Results

**Figure 3:**
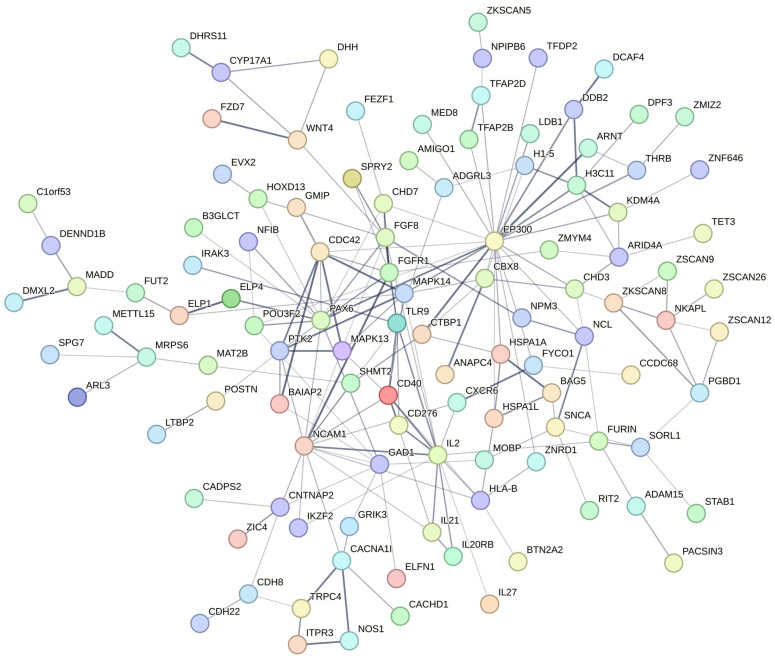
STRING-MODEL of candidate gene list resulted from GWAS meta-meta-analysis showing a fully connected Protein–Protein Interactions (PPIs) network.

Following GWAS meta-meta-analysis, we extracted only the included GWAS from all of the datasets to generate a candidate gene list (combinational gene list). Duplicated genes, pseudogenes, and RNA coding genes were discarded, and remaining genes were checked by a STRING-MODEL of PPIs. Finally, 117 genes from all the significant datasets refined showed a connected network of PPI (Figure 3). The candidate gene list included the following genes: *ZIC4*, *TET3*, *SPG7*, *IKZF2*, *THRB*, *POU3F2*, *BHLHE2*, *CXCR6*, *FURIN*, *FYCO1*, *B3GLCT*, *ELP4*, *PAX6*, *BAIAP2*, *CHD3*, *SNCA*, *GMIP*, *ADGRL3*, *CYP17A1*, *NCL*, *BTN2A2*, *CNTNAP2*, *POLR1H*, *AMIGO1*, *MOBP*, *ZKSCAN8*, *ZSCAN12*, *MED8*, *ZKSCAN5*, *MADD*, *DHRS11*, *LDB1*, *ELP1*, *CTBP1*, *GAD1*, *EP300*, *CBX8*, *RIT2*, *ADAM15*, *TFAP2D*, *ZMIZ2*, *STAB1*, *NKAPL*, *ANAPC4*, *NPIPB6*, *FGF8*, *FZD7*, *BAG5*, *CD276*, *PTK2*, *SORL1*, *CD40*, *CDH22*, *FGFR1*, *POSTN*, *TRPC4*, *NFIB*, *CACNA1I*, *DDB2*, *DMXL2*, *PACSIN3*, *ZMYM4*, *ELFN1*, *SPRY2*, *C1orf53*, *CCDC68*, *DENND1B*, *SHMT2*, *METTL15*, *NCAM1*, *ARL3*, *DHH*, *ITPR3*, *MAT2B*, *CDH8*, *NKAPL*, *ZSCAN26*, *CHD7*, *MRPS6*, *CDC42*, *MAPK13*, *MAPK14*, *WNT4*, *IL20RB*, *TFAP2B*, *CCDC68*, *IL2*, *IL21*, *LTBP2*, *PGBD1*, *CADPS2*, *EVX2*, *FEZF1*, *GRIK3*, *H1-5*, *H3C11*, *HOXD13*, *ZSCAN9*, *TFDP2*, *DCAF4*, *DPF3*, *BARHL2*, *IL27*, *FUT2*, *ARID4A*, *TLR9*, *NOS1*, *CACHD1*, *ZNF646*, *IRAK3*, *HLA-B*, *HSPA1A*, *HSPA1L*, *NPM3*, *KDM4A*, *ZKSCAN8*, and *THSD7A*.

To reach a higher level of assessments, we performed further bioinformatic assessments including systems biology by enrichment analysis (EA) and clustering enrichment analysis (CEA) and in-depth silico analysis by PGx. EA results were accomplished in three main levels including Pathway Analysis, Gene Ontology (GO), and Disease/Drug Assessments (DDAs). Pathway Analysis, based on the Reactome and KEGG databases, revealed that the most significant pathway is Signaling By Receptor Tyrosine Kinases R-HSA-9006934 with a *p*-value of 5.084E−7, q-value (adjusted *p*-value) of 0.0024, and OR of 5.17 (Table 3).

GO Analysis indicated that the top-scored Biological Process resulted from the interaction among the candidate genes is Regulation of Transcription by RNA Polymerase II (GO:0006357) (*p* = 2.65E−10; Q = 3.35E−07; and OR = 4.15), the top-scored Molecular Function is Sequence-Specific DNA Binding (GO:0043565) (*p* = 0.00001; Q = 0.002; and OR = 4.14), and also, the most significant GO for Cellular Component is Nucleus (GO:0005634) (*p* = 0.00015; Q = 0.015; and OR = 2.1) (Table 4).

Finally, DDAs based on DisGeNET and GeDiPNet uncovered that the most plausible disease-causing manifestation resulted from the interaction among the candidate gene list is Autism Spectrum Disorder (ASD) (*p*-value = 1.15E−12; q-value = 3.48E−09; and OR = 8.41). Schizophrenia has the second place with a *p*-value of 2.61E−10, which is significantly weaker than ASD (Table 5).

**Figure 4:**
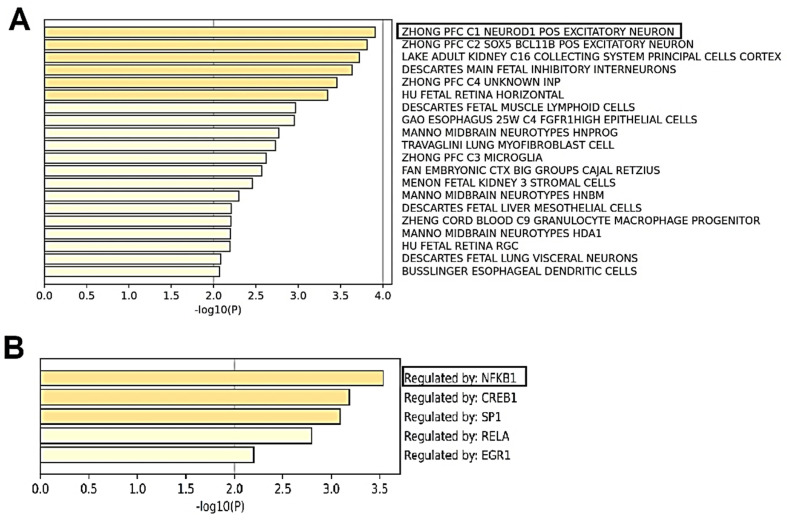
Clustered enrichment analysis based on candidate gene list indicating (**A**) cell type signatures and (**B**) Transcription Factors based on TRRUST.

To obtain a deeper view in our systems biology investigations, we additionally employed Metascape for CEA and found some additional results. Gene list enrichments were identified in the following ontology categories: Cell Type Signatures and TRRUST. All genes in the genome are used as an enrichment background. Terms with a *p*-value lower than 0.01, a minimum count of 3, and an enrichment factor higher than 1.5 were gathered and grouped into clusters based on their membership similarities. An enrichment factor is the ratio between the observed counts and the counts expected by chance. The top few enriched clusters (one term per cluster) are shown in Figure 4A,B. As clear in Figure 4A, ZHONG PFC C1 NEUROD1 POS EXCITATORY NEURON (GO: M39088) was the most significant based on the enrichment analysis in Cell Type Signatures. Notably, Figure 4B indicated that the most significant transcription factor is NFKB1 followed by CREB1 and SP1.

**Figure 5:**
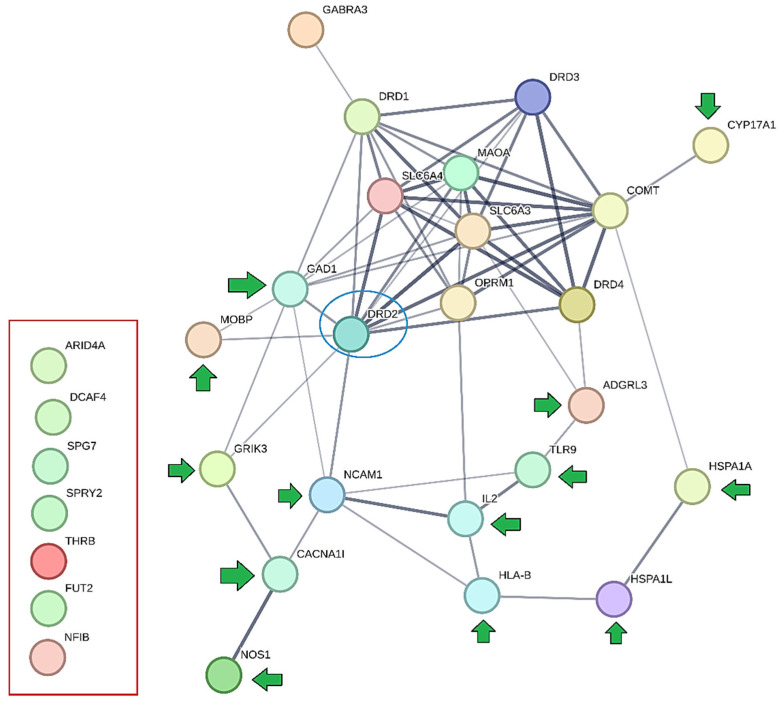
STRING-MODEL of combinational gene list consists of PGx-related genes (Genes with at least one significant variant annotation) and GARS genes. Red rectangle indicates unconnected protein-coding genes; blue circle represents the most potential GARS gene in association with genes from the other group; and green arrows highlight genes from the candidate list with at list a PGx annotation.

Lastly, to suggest potential clinical utility for individuals with combined manifestations of anxiety, depression, and ADHD, we aimed to find the likely candidate Pharmacogenes from the candidate list and tested the PGx involvements of each gene via the PharmGKB database. We then checked all variant annotations of every single gene and found 555 variant annotations in total; among them, 272 variant annotations were statistically significant with at least one variant-disease–drug association. Lack of enough PGx data in the candidate gene list derived from GWAS meta-meta-analysis was a problem. However, we hypothesized a potential solution for it: RDS might be involved, and GARS genes may have connections with the Pharmacogenes of interest. Therefore, we conducted another STRING-MODEL based on the combination of Pharmacogenes (20 genes) with GARS genes (10 genes) (Figure 5). Here is the combinational gene list: *DRD1*, *DRD2*, *DRD3*, *DRD4*, *MAOA*, *COMT*, *SLC6A3*, *SLC6A4*, *OPRM1*, *GABRA3*, *SPG7*, *THRB*, *ADGRL3*, *CYP17A1*, *MOBP*, *GAD1*, *NFIB*, *CACNA1I*, *SPRY2*, *NCAM1*, *IL2*, *GRIK3*, *DCAF4*, *FUT2*, *ARID4A*, *TLR9*, *NOS1*, *HLA-B*, *HSPA1A*, and *HSPA1L.* According to Figure 5, seven genes were not connected with the other genes and the other genes built a fully connected PPI network which means 23 genes were related with each other (13 PGx-related+ 10 GARS). Surprisingly, the most important gene of GARS in association with the PGx-related group was DRD2 with four PPIs with *GAD1, MOBP, GRIK3*, and *NCAM1*. On the other hand, GAD1 showed the highest interactions with GARS genes (*DRD1, DRD2, SLC6A3, SLC6A4, COMT,* and *MAOA*). Also, *GAD1* had interactions with other PGx-related groups including *MOBP, GRIK3,* and *NCAM1*.

**Figure 6:**
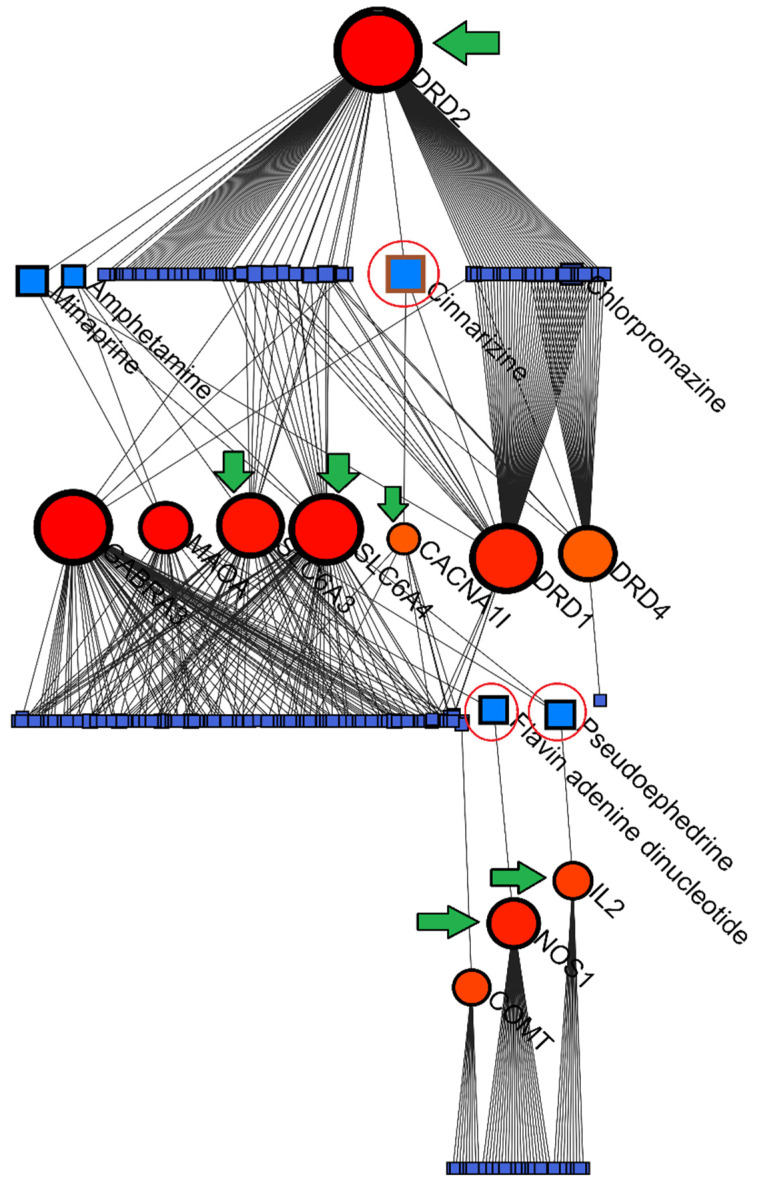
A Protein–Drug interaction (PDI) network generated by NetworkAnalyst in a Sugiyama model indicating potential relationships among PGx-associated gene group (refined primary candidate gene list) and GARS genes. Red circles represent the important drug linker (Cinnarizine) among CACNA1I and DRD1 and DRD2. Green arrows highlight remarkable members.

Finally, to ensure the last part of analysis (including GARS genes) and validate the STRING-MODEL and the whole idea of a RDS solution for the comorbidity of anxiety, depression, and ADHD we performed another in silico prediction. Protein–Drug interactions (PDIs) by NetworkAnalyst provided strong evidenced-based clues for the relationships of final gene list (combinational gene list) containing 23 members (Figure 6). Accordng to the Sugiyama model visualized in Figure 6, Cinnarizine links *CACNA1I* into *DRD2*; Pseudoephedrine links IL2 into *SLC6A3* and *SLC6A4*; and Flavin Adenine dinucleotide links *NOS1* into *MAOA*.

## 6. Discussion

In this study, we first looked into the literature and introduced associations among anxiety, depression, and ADHD; then, with a combinational bioinformatic strategy followed by four separate meta-analyses on GWAS datasets, a new GWAS meta-meta-analysis, systems biology, in-depth silico, and PGx-based analyses, we found Autism Spectrum Disorder (ASD) as the most potential resultant of the interplays among the candidate gene list containing 117 protein-coding genes. All of the results were built upon the GWAS raw data mined from the literature and also the additional PGx guidance were extracted from the PharmGKB database for future personalized medicine treatment and healthcare professional utilizations.

As an interesting part of the analyses, we went beyond the final results and hypothesized a possible solution for the lack of PGx data from the gene list and combined the PGx-related with dopamine homeostasis genes in GARS well-known genes. Collectively, 23 genes remained as the most plausible candidate gene list for comorbidity of anxiety, depression, and ADHD leading to ASD. According to Figure 5, seven genes were not connected with the other genes and the other genes built a fully connected PPI network which means 23 genes were related with each other (13 PGx-related + 10 GARS). Surprisingly, the most important gene of GARS in association with the PGx-related group was DRD2 with four PPIs with *GAD1*, *MOBP*, *GRIK3*, and *NCAM1*. On the other hand, *GAD1* showed the highest interactions with GARS genes (*DRD1*, *DRD2*, *SLC6A3*, *SLC6A4*, *COMT*, and *MAOA*). Also, *GAD1* had interactions with another PGx-related group including *MOBP*, *GRIK3*, and *NCAM1*. This finding agrees with other earlier work by Blum’s group [126].

Since Blum et al. first published in JAMA (1990) concerning the association of the *DRD2* gene polymorphism and severe alcoholism, confirmation has been mixed and controversial. More recently, however, a meta-analysis of 62 studies showed a significant association between *DRD2* rs1800497 and Alcohol Use Disorder (AUD). Other studies from Yale University showed that a haplotype block of the *DRD2* gene A1 allele was associated with AUD and heroin dependence. GWAS studies of depression and suicide in 1.2 million veterans confirmed the first psychiatric candidate gene study finding from Blum et al. 1990; a significant association between the minor *DRD2* allele, Taq A1 (rs1800497 C > T) and severe alcoholism. Additionally, DRD2 rs1800497 is associated with suicide behaviors robustly at *p* = 1.77 × 10^−7^. Furthermore, DNA polymorphic alleles underlying SUD with multiple substances were mapped via chromatin refolding, revealing that the *DRD2* gene and associated polymorphism(s) was the top gene signal (*DRD2*, *p* = 7.9 × 10^−12^). Additionally, based on these investigations, we conclude that GWAS should end the controversy about the *DRD2* gene being at least one determinant of RDS first reported in the Royal Society of Medicine journal 1996 [126].

Moreover, overdose involving opioids is the black heart of the addiction crisis. “Pre-addiction” as an encouraging concept by NIDA and NIAAA, seems best captured with the construct of dopamine dysregulation. Referring to the abundant publications on “reward deficiency syndrome” (RDS), Genetic Addiction Risk Score (GARS) test, RDSQ29, and KB220, pre-addiction can be referred to as “reward dysregulation” as a suitable suggestion. The hypothesis is that the true phenotype is RDS, and other behavioral disorders are endophenotypes where the genetic variants play important roles specifically in the Brain Reward Cascade (BRC). In unpublished but presented study our group tested the pharmacogenomics of the GARS panel by a multi-model in silico investigation in four layers: (1) Protein–Protein Interactions (PPIs); (2) Gene Regulatory Networks (GRNs); (3) disease, drugs, and chemicals (DDCs); and (4) Gene Co-Expression Networks (GCNs). Specifically, all in silico findings were combined together in an enrichment analysis for 59 refined genes which represented highly significant associations of dopamine pathways in the BRC and supported our hypothesis. These results provide scientific evidence for the importance of incorporating GARS as a predictive test to identify pre-addiction, to introduce unique therapeutic targets assisting in the treatment of pain, drug dosing of prescription pharmaceuticals, and identify the risk for subsequent addiction early in -life.

According to Figure 6 and together with the results of Figure 5 (PPIs in STRING-MODEL), we can conclude these possibilities:

(1) *CACNA1I* has a direct linkage (PDI: Figure 6) and an indirect linkage (PPI: Figure 5) with DRD2; thus, NCAM1 can be a highly potential candidate for future PGx studies which links ASD with dopamine homeostasis.
(2) IL2 has a direct linkage (PDI: Figure 6) and indirect linkages (PPI: Figure 5) with *SLC6A3* and *SLC6A4*; thus, OPRM*1* can be a potential candidate for having PGx with ASD and dopamine homeostasis mediated in-art by both the serotonin and dopamine transporter systems.
(3) *NOS1* has a direct linkage (PDI: Figure 6) and long indirect linkages (PPI: Figure 5) with *MAOA*; thus, the following PPI line can be considered for future studies: *NOS1-CACNA1I-NCAM1-DRD2-MAOA*.

Obviously, we highly recommend further experimental and clinical validations due to the complex nature of psychological disorders and unknown future findings. In light of our robust findings, we have carefully discussed the current treatment options regarding comorbidity of anxiety, depression, and ADHD.

## 7. Summary

We are proposing herein that futuristic smart non-addicting treatment should consider early identification of genetic antecedents to ADHD and RDS and incorporate the concepts related to induction of dopamine homeostasis by utilizing the GARS to identify risk polymorphisms coupled with KB220 along with behavioral therapies (e. g Mindfulness, AI etc.).

## 8. Conclusions

Approximately one in five children in the United States suffers from some form of mental illness with multiple comorbidities. These comorbidities, such as depression and anxiety, lead to poor general health. It is well-established that comorbidities are caused by both DNA antecedents as well as epigenetic insults. ADHD specifically is one of the most common intellectual disabilities faced by both children and adults today. ADHD is characterized by core symptoms of inattention, impulsiveness, and hyperactivity with additional symptoms of emotional dysregulation. It is noteworthy that there is emerging genetic and epigenetic evidence which indicate shared common genetic variants across the brain reward circuitry and specific epigenetic insults. Unfortunately, over 60% of adolescents in community-based substance use disorder treatment programs also meet diagnostic criteria for another mental illness.

## Figures and Tables

**Table 1:** Details of meta-analysis, meta-meta-analysis, systems biology, and PGx methods performed in this study.

Identifier	Database	Site	Software (Version)	References [DOI] and Ref (Number)
GWAS data mining	GWAS catalog	https://www.ebi.ac.uk/gwas/home; accessed 3 November 2024	EMBL-EBI 2024	10.1093/nar/gkac1010 [117]
CMA	CMA3	https://meta-analysis.com/pages/video_cma4; accessed 3 November 2024	CMA3 (3.3.070)	10.3390/pharmacy11060182 [118]
PPIs	STRING-MODEL	https://string-db.org/; accessed 3 November 2024	STRING (12.0)	10.3390/jpm13081201 [119]
EA	Pathway Analysis	https://maayanlab.cloud/Enrichr/; accessed 3 November 2024	Enrichr	10.1038/s41398-022-02069-8 [120]
	GO	https://maayanlab.cloud/Enrichr/; accessed 3 November 2024	Enrichr	10.3389/fneur.2022.1077178 [121]
	DDA	https://maayanlab.cloud/Enrichr/; accessed 3 November 2024	Enrichr	10.1080/07391102.2023.2191719 [122]
MA	CEA	https://metascape.org/gp/index.html#/main/step1; accessed 3 November 2024	Metascape	10.1038/s41467-019-09234-6 [123]
PGx	VAA	https://www.pharmgkb.org/; accessed 3 November 2024	PharmGKB	10.3390/jpm13111550 [124]
PDIs	Drug Bank	https://go.drugbank.com/; accessed 3 November 2024	NetworkAnalyst (3.0)	10.1186/1471-2164-14-S4-S1 [125]

Abbreviations are follows: GWAS: Genome-Wide Association Studies; PPIs: Protein–Protein Interactions; Networks; EA: enrichment analysis; GO: Gene Ontology; DDA: Disease/Drug Assessment; MA: meta-analysis; CEA: clustering enrichment analysis; PGx: pharmacogenomics: VAA: Variant Annotation Assessment.

**Table 2:** Refined data for Meta1, Meta2, Meta3, and Meta4 datasets containing all required details for CMA.

No.	ID	PubMed ID	First Author	Best *p*-Value	Sample Size	Ethnicity
1	Meta1	29942085	Nagel 2018	1.00E−19	348,219	European
2	Meta1	34734193	Sun 2020	2.00E−10	20,863	African American + European + Asian + Hispanic
3	Meta1	34118634	Chu 2021	9.00E−09	15,385	British
4	Meta1	34634379	Mei 2021	4.00E−11	74,345	European
5	Meta1	34684344	Zhang 2021	2.00E−10	120,590	European
6	Meta1	37029353	Jia 2023	6.00E−10	83,615	British
7	Meta1	37106081	Schoeler 2023	4.00E−09	101,859	European
8	Meta1	38328521	Yakovchik 2024	1.00E−11	30,063	Russian
9	Meta2	19165232	Otowa 2009	4.00E−09	400	Japanese
10	Meta2	23726511	Xie 2013	3.00E−09	5218	African American + European
11	Meta2	25456346	Nievergelt 2014	2.00E−09	3494	European, Hispanic + Native American + African American + East Asian
12	Meta2	26754954	Otowa 2016	2.00E−08	21,761	European
13	Meta2	27167565	Stein 2016	6.00E−08	7777	African American + Latino American + European
14	Meta2	30456828	Khramtsova 2018	6.00E−08	9870	European
15	Meta2	31116379	Meier 2019	1.00E−11	29,536	European
16	Meta2	31594949	Nievergelt 2019	3.00E−09	195,701	European + African + Latino + Native American
17	Meta2	31619474	Zhu 2019	4.00E−08	411,593	European
18	Meta2	31748690	Purves 2019	3.00E−11	83,566	European
19	Meta2	31835028	Psychiatric Genomics Consortium 2019	1.00E−27	727,036	European
20	Meta2	31906708	Levey 2020	2.00E−08	224,330	African American + European
21	Meta2	32231276	Cai 2020	7.00E−22	274,107	British
22	Meta2	33510476	Stein 2021	3.00E−10	214,408	European
23	Meta2	33686288	Peyrot 2021	1.00E−08	7507	European
24	Meta2	33893285	Guindo-Martinez 2021	5.00E−08	56,637	European
25	Meta2	34865855	Maihofer 2021	2.00E−11	217,491	European
26	Meta2	35026594	Li 2022	2.00E−08	38,670	British
27	Meta2	35181757	Wendt 2022	1.00E−19	497,803	European
28	Meta2	36753304	Gong 2023	4.00E−08	630,986	European
29	Meta2	37164147	Li 2023	1.00E−09	502,656	British
30	Meta2	37218628	Zhou 2023	2.00E−10	174,659	European
31	Meta2	38043635	Dai 2023	5.00E−12	9725	European
32	Meta3	32724131	Li 2020	4.00E−08	255	European
33	Meta3	33479212	Yao 2021	2.00E−22	500,199	European
34	Meta3	33483693	Clements 2021	6.00E−10	5086	European
35	Meta3	33893285	Guindo-Martinez 2021	3.00E−09	56,637	European
36	Meta3	34159505	Wang 2021	2.00E−18	119,754	European
37	Meta3	34634379	Mei 2021	3.00E−11	42,455	European
38	Meta3	36324662	Suppli 2021	2.00E−08	38,716	European
39	Meta3	34859065	Zhang 2021	1.00E−11	927,055	European
40	Meta3	35898629	Yin 2022	2.00E−19	829,870	
41	Meta3	36228427	Yuan 2022	2.00E−08	724	Han Chinese
42	Meta3	36672180	Tirozzi 2023	6.00E−19	807,553	European
43	Meta3	37426090	Li 2023	1.00E−12	91,643	European
44	Meta3	37390107	Baltramonaityte 2023	7.00E−74	562,507	European + South Asian + East Asian + Hispanic + African American
45	Meta3	38177345	Meng 2024	2.00E−27	1,820,689	European + African + East Asian + South Asian + Hispanic + Latin American
46	Meta3	38858783	Yu 2024	3.00E−43	633,531	UK
47	Meta4	18839057	Lesch 2008	1.00E−08	593	European
48	Meta4	23453885	Smoller 2013	2.00E−12	61,220	European
49	Meta4	23728934	Yang 2013	6.00E−09	2003	Han Chinese
50	Meta4	27890468	van Hulzen 2016	2.00E−08	28,139	European
51	Meta4	28416812	Yang 2017	5.00E−09	780	Han Chinese
52	Meta4	29325848	Martin 2017	2.00E−08	32,102	European
53	Meta4	32595297	Kweon 2018	1.00E−08	27	Korean
54	Meta4	29769613	Sun 2018	3.00E−08	547	East Asian
55	Meta4	30289880	Qi 2018	2.00E−08	54,230	UK
56	Meta4	30478444	Demontis 2018	4.00E−10	55,374	European + Han Chinese
57	Meta4	30563984	Hawi 2018	3.00E−08	1688	European
58	Meta4	30610198	Soler Artigas 2019	3.00E−11	83,129	European
59	Meta4	30818988	Klein 2019	1.00E−10	79,398	European
60	Meta4	31619474	Zhu 2019	5.00E−18	447,576	European
61	Meta4	31835028	Psychiatric Genomics Consortium 2019	1.00E−27	497,807	European
62	Meta4	32279069	Rovira 2020	2.00E−08	49,560	European
63	Meta4	32606422	Wu 2020	8.00E−14	61,421	European
64	Meta4	33479212	Yao 2021	5.00E−19	728,648	European
65	Meta4	33495439	Demontis 2021	3.00E−10	36,430	European
66	Meta4	33686288	Peyrot 2021	3.00E−17	59,774	UK
67	Meta4	34154395	Brikell 2021	5.00E−08	4991	European
68	Meta4	34446935	Karlsson Linner 2021	7.00E−59	2,776,348	European
69	Meta4	35717853	Baranova 2022	4.00E−09	101,724	European
70	Meta4	35764056	Rao 2022	4.00E−42	371,591	European
71	Meta4	35927488	Rajagopal 2022	2.00E−11	53,181	Danish
72	Meta4	36753304	Gong 2023	1.00E−11	509,620	European
73	Meta4	37689771	Pedersen 2023	2.00E−14	58,286	European
74	Meta4	38565336	Chen 2024	3.00E−36	730,796	European

Meta1 refers to anxiety, and Meta2, Meta3, and Meta4 were anxiety disorder, depressive disorder, and ADHD, respectively.

**Table 3:** Pathway Analysis of candidate gene list based on Reactome and KEGG databases.

Index	Name	*p*-Value	q-Value	OR
Reactome	Signaling By Receptor Tyrosine Kinases R-HSA-9006934	5.08E−06	0.002373	5.17
Reactome	Netrin-1 Signaling R-HSA-373752	8.6E−06	0.002373	20.69
Reactome	VEGFA-VEGFR2 Pathway R-HSA-4420097	1.53E−05	0.002815	12.64
Reactome	Signaling By VEGF R-HSA-194138	2.59E−05	0.003578	11.45
Reactome	Activation Of TFAP2 (AP-2) Family Of Transcription Factors R-HSA-8866907	3.82E−05	0.004219	59.69
KEGG	Proteoglycans in cancer	2.31E−05	0.004342	7.54
KEGG	Yersinia infection	0.000135	0.01067	8.38
KEGG	Signaling pathways regulating pluripotency of stem cells	0.00017	0.01067	8.01
KEGG	Lipid and atherosclerosis	0.000234	0.01067	6.19
KEGG	MAPK signaling pathway	0.000284	0.01067	5.17
KEGG	VEGF signaling pathway	0.000358	0.01121	13.11
KEGG	Toxoplasmosis	0.000455	0.01222	8.48
KEGG	Growth hormone synthesis, secretion and action	0.0006	0.01411	7.96
KEGG	Adherens junction	0.000725	0.01514	10.76
Reactome	Gene Expression (Transcription) R-HSA-74160	0.000187	0.01723	2.75
KEGG	Pathways in cancer	0.000922	0.01734	3.57
Reactome	Disease R-HSA-1643685	0.000289	0.02275	2.54
Reactome	SUMO E3 Ligases SUMOylate Target Proteins R-HSA-3108232	0.000405	0.02352	6.76
Reactome	Generic Transcription Pathway R-HSA-212436	0.000407	0.02352	2.8
Reactome	Attenuation Phase R-HSA-3371568	0.000426	0.02352	23.34

q-value and OR refer to adjusted *p*-value and Odds Ratio, respectively.

**Table 4:** Gene Ontology (GO) assessments according to the candidate gene lists through the Biological, Cellular, and Molecular categorizations.

Index	Name	*p*-Value	q-Value	OR
GO Biological Process	Regulation Of Transcription By RNA Polymerase II (GO:0006357)	2.65E−10	3.35E−07	4.15
GO Biological Process	Regulation Of DNA-templated Transcription (GO:0006355)	4.41E−09	2.79E−06	3.88
GO Biological Process	Negative Regulation Of Cellular Component Organization (GO:0051129)	3.35E−07	0.000141	18
GO Molecular Function	Sequence-Specific DNA Binding (GO:0043565)	1.39E−05	0.001911	4.14
GO Molecular Function	RNA Polymerase II Transcription Regulatory Region Sequence-Specific DNA Binding (GO:0000977)	1.82E−05	0.001911	3.3
GO Biological Process	Regulation Of Interleukin-12 Production (GO:0032655)	9.51E−06	0.003006	20.23
GO Molecular Function	Sequence-Specific Double-Stranded DNA Binding (GO:1990837)	5.64E−05	0.003322	3.83
GO Molecular Function	RNA Polymerase II Cis-Regulatory Region Sequence-Specific DNA Binding (GO:0000978)	6.33E−05	0.003322	3.19
GO Molecular Function	DNA Binding (GO:0003677)	9.21E−05	0.003869	3.47
GO Biological Process	Regulation Of Gene Expression (GO:0010468)	1.95E−05	0.004487	3.39
GO Biological Process	Positive Regulation Of B Cell Proliferation (GO:0030890)	2.13E−05	0.004487	28.89
GO Molecular Function	Cis-Regulatory Region Sequence-Specific DNA Binding (GO:0000987)	0.000159	0.00555	3.05
GO Biological Process	Generation Of Neurons (GO:0048699)	5.85E−05	0.00911	7.82
GO Biological Process	Neuron Differentiation (GO:0030182)	6.07E−05	0.00911	7.77
GO Biological Process	Positive Regulation Of Lymphocyte Proliferation (GO:0050671)	6.49E−05	0.00911	13.17
GO Molecular Function	Double-Stranded DNA Binding (GO:0003690)	0.000337	0.01012	3.55
GO Biological Process	Positive Regulation Of Transcription By RNA Polymerase II (GO:0045944)	8.31E−05	0.0105	3.36
GO Cellular Component	Nucleus (GO:0005634)	0.000156	0.01515	2.1
GO Cellular Component	Intracellular Membrane-Bounded Organelle (GO:0043231)	0.000242	0.01515	2.02
GO Molecular Function	Zinc Ion Binding (GO:0008270)	0.000756	0.01983	4.43
GO Molecular Function	Transition Metal Ion Binding (GO:0046914)	0.001206	0.02815	3.73
GO Molecular Function	Inositol 1,4,5 Trisphosphate Binding (GO:0070679)	0.001407	0.02954	44.37
GO Cellular Component	Calcium Channel Complex (GO:0034704)	0.004649	0.1828	9.57
GO Cellular Component	Axon (GO:0030424)	0.006421	0.1828	4.52
GO Cellular Component	Cytoskeleton (GO:0005856)	0.007312	0.1828	2.8
GO Cellular Component	Catenin Complex (GO:0016342)	0.01105	0.2006	13.64
GO Cellular Component	Golgi Membrane (GO:0000139)	0.01123	0.2006	3.03
GO Cellular Component	Neuron Projection (GO:0043005)	0.01444	0.2256	2.66
GO Cellular Component	Cul4-RING E3 Ubiquitin Ligase Complex (GO:0080008)	0.01695	0.2327	10.74
GO Cellular Component	Bounding Membrane Of Organelle (GO:0098588)	0.01861	0.2327	2.27

q-value and OR indicate adjusted *p*-value and Odds Ratio, respectively.

**Table 5:** Drug/Disease Assessments (DDAs) of candidate gene list according to the latest updates of DisGeNET and GeDiPNet databases.

Index	Name	*p*-Value	q-Value	OR
DisGeNET	Autism Spectrum Disorder	1.15E−12	3.48E−09	8.41
DisGeNET	Schizophrenia	2.61E−10	3.94E−07	4.22
DisGeNET	Infantile uterus	1.45E−09	1.46E−06	43.3
GeDiPNet	Schizophrenia	1.01E−08	7.01E−06	4.16
GeDiPNet	Anxiety Disorder	4.56E−08	1.59E−05	7.3
GeDiPNet	Mental Depression	2.46E−07	5.71E−05	4.15
GeDiPNet	Mirror Movements	9.81E−07	0.000147	33.74
GeDiPNet	Hypopituitarism	1.06E−06	0.000147	20.79
GeDiPNet	Paraplegia	2.38E−06	0.000276	27.6
GeDiPNet	Hypospadias	3.97E−06	0.000395	7.12
DisGeNET	Primary physiological amenorrhea	5.55E−07	0.000419	16.61
GeDiPNet	Physiological Amenorrhea	5.08E−06	0.000442	11.65
DisGeNET	Alcoholic Intoxication, Chronic	9.06E−07	0.000547	6.1
GeDiPNet	Cryptorchidism	0.000012	0.000909	4.45
GeDiPNet	Lupus Erythematosus	1.31E−05	0.000909	6.17
DisGeNET	Sense of smell impaired	2.72E−06	0.001192	26.78
DisGeNET	Graves Disease	3.42E−06	0.001192	6.45
DisGeNET	B-Cell Lymphomas	3.88E−06	0.001192	4.38
DisGeNET	Lymphoma	3.96E−06	0.001192	3.46
DisGeNET	ADHD	4.14E−06	0.001192	5.73

Abbreviations: ADHD: attention deficit hyperactivity disorder.

## Data Availability

The original contributions presented in this study are included in the article/Supplementary Materials. Further inquiries can be directed to the corresponding authors.

## Supplementary Materials

The following supporting information can be downloaded at: https://www.mdpi.com/article/10.3390/jpm15030103/s1, Figure S1: Results of Meta-analysis for Meta1 dataset; Figure S2: Results of Meta-analysis for Meta2 dataset; Figure S3: Results of Meta-analysis for Meta3 dataset; Figure S4: Results of Meta-analysis for Meta4 dataset; Figure S5: Funnel Plot for publication bias of Meta1; Figure S6: Funnel Plot for publication bias of Meta2; Figure S7: Funnel Plot for publication bias of Meta3; Figure S8: Funnel Plot for publication bias of Meta4.
