# Supplementary material for: Genetic and Regulatory Mechanisms of Comorbidity of Anxiety, Depression and ADHD: A GWAS Meta-Meta-Analysis Through the Lens of a System Biological and Pharmacogenomic Perspective in 18.5 M Subjects
Source: J Pers Med. 2025 Mar 5;15(3):103. doi: 10.3390/jpm15030103 (PMC11943124; doi:10.3390/jpm15030103)

Figure S1: Results of Meta-analysis for Meta1 dataset.

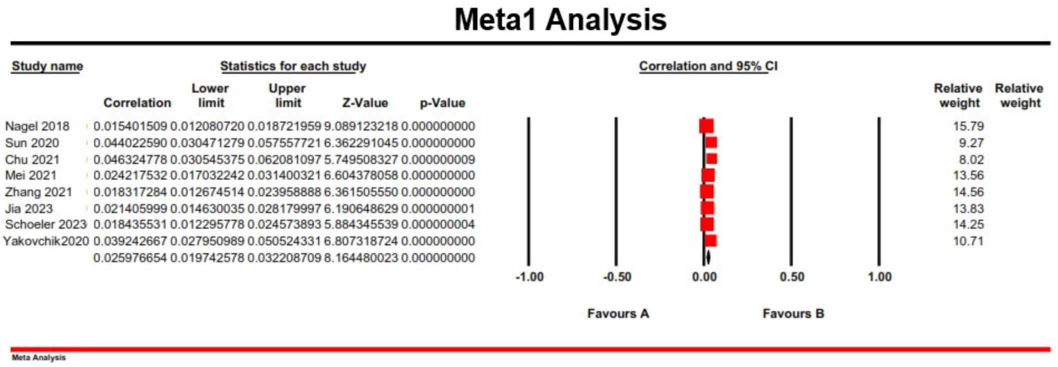

Figure S2: Results of Meta-analysis for Meta2 dataset.

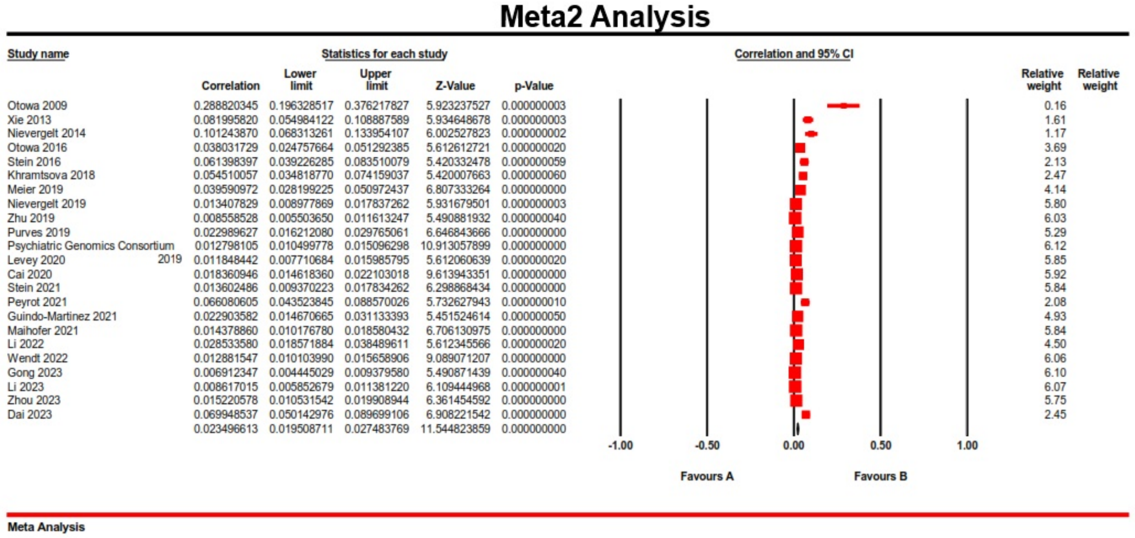

Figure S3: Results of Meta-analysis for Meta3 dataset.

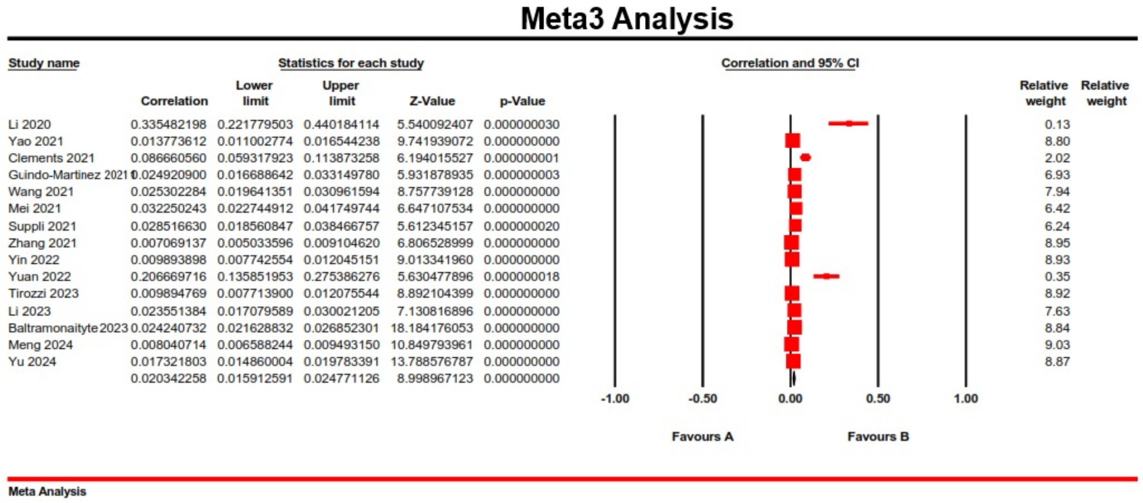

Figure S4: Results of Meta-analysis for Meta4 dataset.

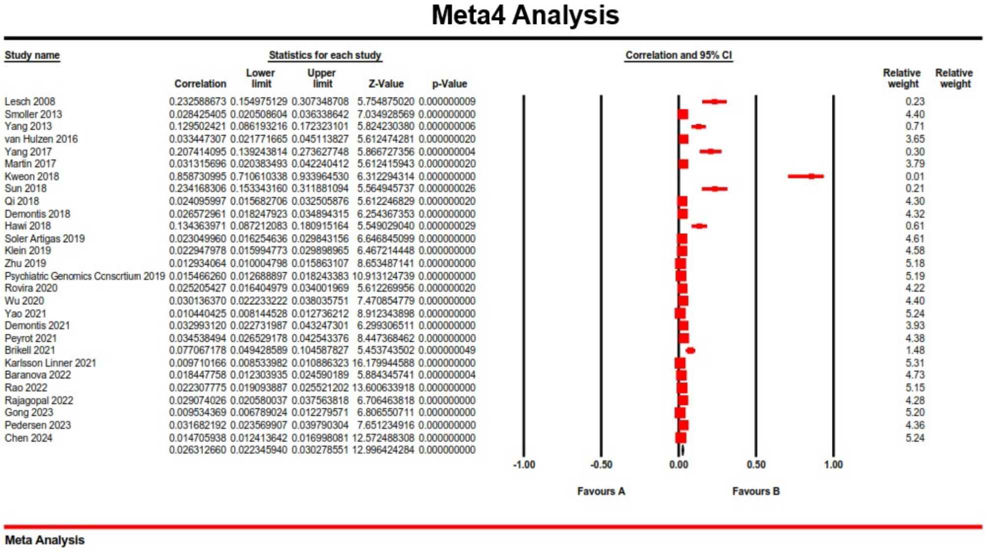

Figure S5: Funnel Plot for publication bias of Meta1.

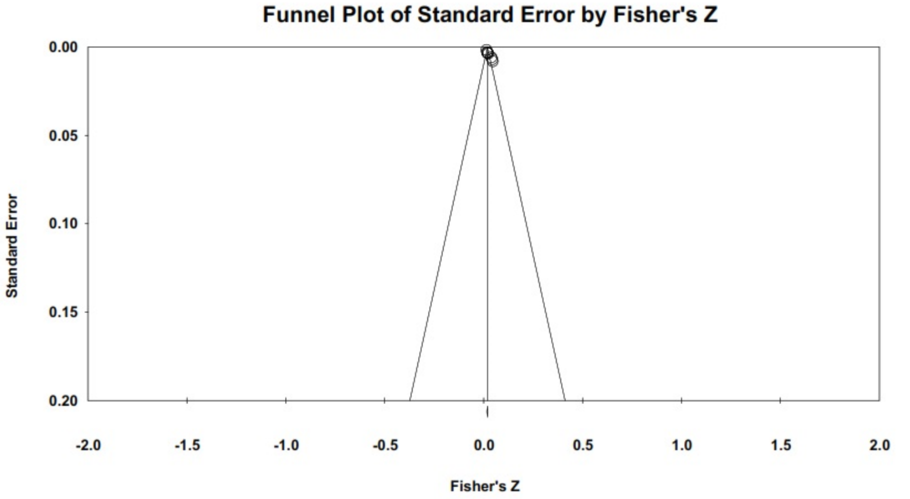

Figure S6: Funnel Plot for publication bias of Meta2.

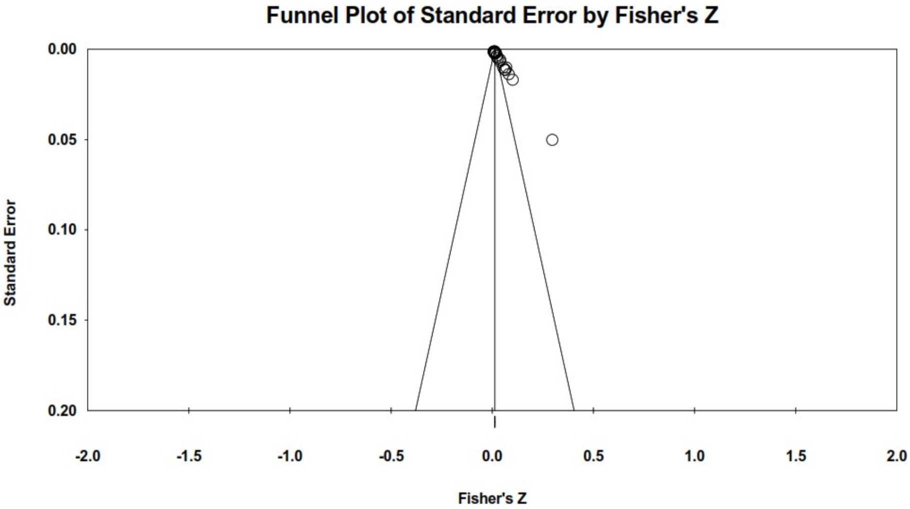

Figure S7: Funnel Plot for publication bias of Meta3.

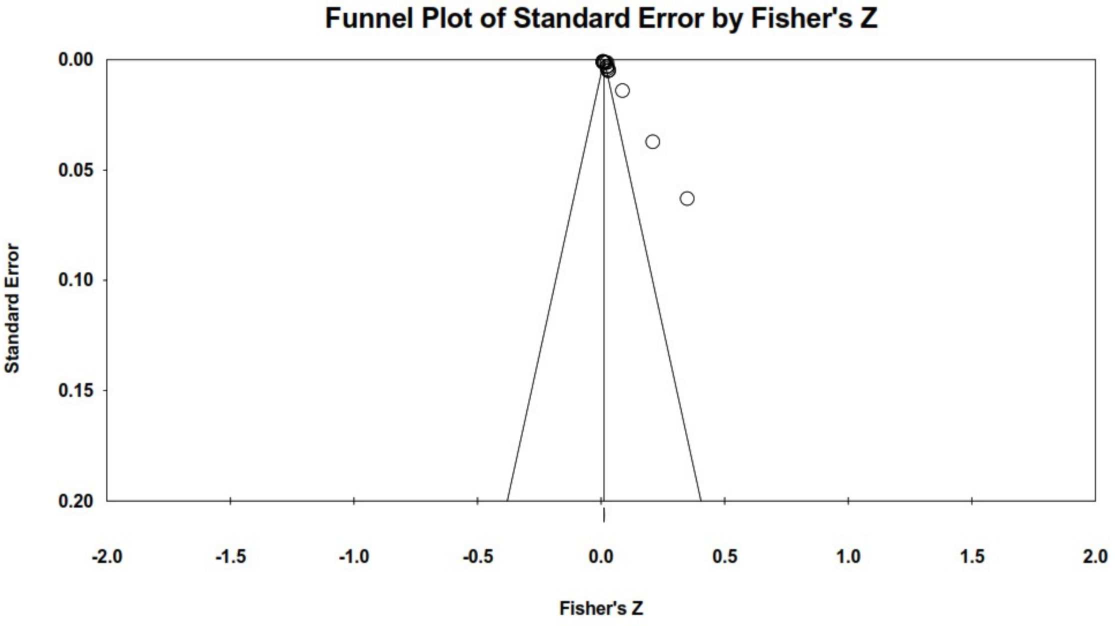

Figure S8: Funnel Plot for pub-lication bias of Meta4.

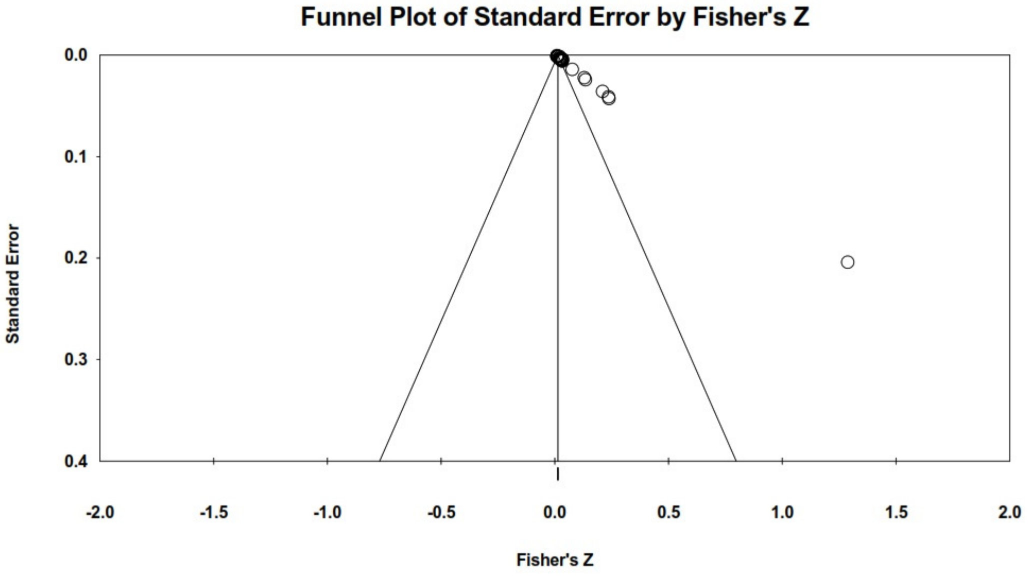

Supplement: Supplementary file 1 [file jpm-15-00103-s001.zip › jpm-3370522-supplementary.pdf]
